# Assessment of PFAS toxicity using *lux*-marked bioluminescent *Pseudomonas* biosensors

**DOI:** 10.1093/femsle/fnag135

**Published:** 2026-10-30

**Authors:** Diana Senovilla-Herrero, Katrina Campbell, Sarah Helyar, Heather Moore, Lenka Mbadugha

**Affiliations:** Institute for Global Food Security, School of Biological Sciences, Queen’s University Belfast, 19 Chlorine Gardens, Belfast BT9 5DL, United Kingdom; Institute for Global Food Security, School of Biological Sciences, Queen’s University Belfast, 19 Chlorine Gardens, Belfast BT9 5DL, United Kingdom; Institute for Global Food Security, School of Biological Sciences, Queen’s University Belfast, 19 Chlorine Gardens, Belfast BT9 5DL, United Kingdom; Agri-Food and Biosciences Institute, 18a Newforge Lane, Belfast BT9 5PX, United Kingdom; School of Biological Sciences, University of Aberdeen, St Machar Drive, Aberdeen AB24 3UU, United Kingdom

**Keywords:** *Pseudomonas putida* F1, *Pseudomonas putida* 12708, per- and polyfluoroalkyl substances (PFAS), biosensors, bioluminescence, fluoride toxicity

## Abstract

Per- and polyfluoroalkyl substances (PFAS) are persistent fluorinated contaminants of growing environmental concern, yet rapid methods for assessing their biological effects remain limited. This study evaluated *lux*-marked *Pseudomonas putida* F1 Tn5 *lux*CDABE and *P. putida* 12 708 Tn5 *lux*CDABE as whole-cell biosensors for detecting sublethal PFAS toxicity. Fluoride bioassays confirmed both strains tolerated up to 1000 mg l^−1^ fluoride without measurable bioluminescence inhibition, supporting their suitability for PFAS exposure studies. Growth and bioluminescence responses were then assessed in M9 minimal medium following exposure to PFOA, PFNA, and PFHxS. Conventional growth measurements showed limited effects across tested concentrations, with strain-specific reductions observed mainly in *P. putida* F1 Tn5 *lux*CDABE. Bioluminescence revealed clearer compound- and concentration-dependent responses. PFOA and PFNA caused significant inhibition of peak bioluminescence in *P. putida* F1 Tn5 *lux*CDABE, while *P. putida* 12 708 Tn5 *lux*CDABE showed a variable response, including increased luminescence following PFOA exposure and inhibition at higher concentrations of selected PFAS. These findings demonstrate that *lux*-marked *P. putida* biosensors are able to detect PFAS-induced metabolic stress with greater sensitivity than conventional growth assays and highlights their potential as rapid tools for ecotoxicological screening of fluorinated contaminants.

## Teaser

Bioluminescent *Pseudomonas* biosensors rapidly detected metabolic stress caused by PFAS pollutants, revealing toxic inhibitory effects and supporting their use for environmental screening.

## Introduction

Per- and polyfluoroalkyl substances (PFAS) encompass over 10 000 synthetic fluorinated compounds characterized by exceptional thermal and chemical stability (Senovilla-Herrero et al. 2024). Their resistance to degradation and amphiphilic properties drive both persistence and bioaccumulation in the ecosystems (Zeeshan et al. 2025). Recent advances in PFAS state-of-the-art analytical methods, i.e. liquid chromatography-tandem mass spectrometry (LC-MS/MS), have greatly improved the detection and quantification of PFAS compounds. However, these techniques are still labour-intensive, costly, expertise-dependent, and time-consuming. Moreover, because they rely on available analytical standards for targeted compound detection, they do not capture the associated biological or ecotoxicological effects of the compounds nor account for pollutant bioavailability (Weitz 2000, Dévier et al. 2011). Biological assays and associated ecotoxicity tests are able to provide complementary information to analytical techniques by providing a biological measure of contamination (Hassan et al. 2016). Microbial biosensors have been widely used in ecotoxicity testing with the purpose of *in situ* environmental management (Shin 2011). Whole cell bioluminescence biosensors, in particular, offer the advantage of visible light emission as the sensor signal transduction (Weitz 2000, Philp et al. 2004). As bioluminescence is a direct cause of intracellular biochemical reactions, metabolically active cells are the only bacterial cells able to luminesce. Therefore, exposure to increased concentrations of environmental contaminants will compromise the bacterial cell metabolism, impairing their ability to emit light. Consequently, bioluminescent bacteria have been used as biosensors to measure chemical contamination, and in turn, environmental stress, in the form of decreasing light output (Weitz 2000, Philp et al. 2004, Fernández-Piñas et al. 2014). Genetically modified bioluminescent biosensors have previously been developed by introducing the mini-Tn5 transposon, carrying the *Aliivibrio fischeri* luxCDABE genes responsible for light production, into environmentally relevant *Pseudomonas* strains via biparental mating, resulting in stable chromosomal integration (Weitz et al. 2001).

*Pseudomonas fluorescens* 8866 Tn5 *lux*CDABE and *Pseudomonas putida* F1 Tn5 *lux*CDABE strains, e.g. have been used to assess the toxicity of a range of environmental organic pollutants including toluene, benzene, 3,5-dichlorophenol, as well as co-contaminant metals copper and zinc in groundwater samples (Paton et al. 1995, Preston et al. 2000, Weitz et al. 2001, Tiensing et al. 2002, Bhattacharyya et al. 2005, Zhang et al. 2016). Other GM *lux*-marked *P. putida* strains have also been previously used to assess the toxicity of complex hydrocarbon mixtures (Selifonova and Eaton 1996), silver, zinc oxide and titanium dioxide nanoparticles (Dams et al. 2011, Mallevre et al. 2014), petroleum (Maciel 2003), and heavy metals in contaminated soils (Fernández-Piñas et al. 2014). Nonetheless, studies focusing on PFAS toxicity remain limited.

PFAS toxicity in bacteria is primarily associated with membrane disruption, oxidative stress, and morphological changes (Smorada et al. 2024). These compounds can penetrate lipid bilayers and alter membrane fluidity, with longer fluorinated chains and sulfonate head groups causing greater membrane disruption (Liu et al. 2016). They also induce stress responses, such as reactive oxygen species production and extracellular polymeric substance (EPS) formation (LaFond et al. 2023). Mechanisms of toxicity vary depending on PFAS structure and microbial physiology. For instance, Liu et al. (2016) differentiated between perfluorooctanesulfonic acid (PFOS) (C_8_F_17_SO_3_H) predominantly disrupting membrane fluidity and perfluorooctanoic acid (PFOA) (C_8_HF_15_O_2_) causing oxidative DNA damage. Short-chain PFAS, including perfluorohexane sulfonate (PFHxS) (C_6_F_13_SO_3_H), are of growing concern because they are increasingly replacing long-chain compounds like PFOA and PFOS. Yet, they represent terminal biotransformation products of longer PFAS that are themselves resistant to further breakdown (Presentato et al. 2020), and their effects on microbial receptors remain poorly understood.

*Pseudomonas* species are frequently associated with PFAS-contaminated environments (Smorada et al. 2024). While some strains have been reported to utilize PFAS as a carbon source, complete mineralization remains rare (Yi et al. 2019, Presentato et al. 2020). A key constraint is the toxicity of released fluoride (F^−^) ions, which inhibit essential enzymes (Wackett 2022). A number of *Pseudomonas* species, however, have been reported to mitigate fluoride stress through adaptive mechanisms. These include efflux systems, such as the CrcB fluoride ion exporter, and other broader metabolic responses (Chetverikov et al. et al. 2023, Smorada et al. 2024), which may support survival in PFAS-contaminated environments.

Select *Pseudomonas* strains have been proven to transform PFAS compounds, although the extent and mechanisms of degradation remains variable. These degradation studies also provide indirect insight into toxicity constraints, as inhibitory effects and concentration-dependent performance are often reflected in transformation efficiency. Kwon et al. (2014) isolated *P. aeruginosa* strain HJ4 and reported up to 67% of PFOS biodegradation within 47 h at initial concentrations of 1.4–1.8 mg l^−1^. Chetverikov et al. (2017) described complete PFOS biodegradation by isolated strain *P. plecoglossicida* 2.4-D at a concentration of 1 g l^−1^ in liquid medium within 6 days, and 75% degradation in soil in 6 months. Yi et al. (2019) showed *P. parafulva* YAB-1 was able to degrade PFOA by 34.2% in 4 days in media containing glucose supplementation. Chiriac et al. (2023) compared PFOA and PFOS degradation by *P. putida* and *P. aeruginosa* species at various concentrations up to 10 mg l^−1^ over 4 days. Findings demonstrated partial transformation rates and by-products differed by strain, with efficiency being inversely proportional to concentration, suggesting concentration-dependent inhibitory or toxic effects. Chetverikov et al. (2023) identified *P. mosselii* strain 5(3) as the most versatile *Pseudomonas* degrader reported to date. Its fully sequenced genome contains a unique suite of degradation genes capable of utilizing C_7_-C_10_ perfluorocarboxylic acids (PFCAs) at a concentration of 250 mg l^−1^ within 7 days, reflecting substantial physiological tolerance, with degradation performance likely constrained by toxic stress at elevated PFAS concentrations.

Despite advances in understanding PFAS and microbe interactions, significant knowledge gaps remain. In particular, while luminescence-based assays have demonstrated sensitivity to PFAS-induced stress in model organisms, such as *A. fischeri* (Fitzgerald et al. 2018), the use of *lux*-based *Pseudomonas* biosensors to quantify sublethal toxicity of individual PFAS compounds under controlled conditions has not been widely explored. Given the ecological relevance and metabolic versatility of *P. putida* strains, such systems offer potential for more informative and environmentally relevant toxicity assessment. To address this knowledge gap, the aim of this study was to evaluate *lux*-marked *Pseudomonas* biosensors as tools for detecting sublethal PFAS toxicity by characterizing their growth and bioluminescence responses to fluoride and individual PFAS compounds (PFOA, PFNA, and PFHxS), and to compare the sensitivity of these bioluminescence-based assays with conventional growth measurements.

## Materials and methods

### Materials and equipment

Agar (bacteriological and purified) and quarter-strength Ringer’s solution were purchased from Oxoid Ltd. (Basingstoke, UK). Lysogeny broth (LB) (Lennox) and kanamycin antibiotic (monosulphate salt) were purchased from Sigma–Aldrich (Dorset, UK). BD Difco™ M9 Minimal Salts 5X were purchased from Davidson & Hardy Ltd. (Belfast, UK) (Table 1). D-glucose and potassium chloride were purchased from Fisher Scientific (Loughborough, UK). Magnesium sulphate heptahydrate was purchased from Thermo Scientific (Loughborough, UK). PFOS, perfluorononanoic acid (PFNA), and perfluorohexanesulfonic acid (PFHxS) were purchased from LGC Standards, UK (Middlesex, UK). PFOA was purchased from Thermo Fisher Scientific, UK. Sodium fluoride was purchased from Sigma–Aldrich (Dorset, UK).

**Table 1:** Growth media components.

Lysogeny broth (Lennox)	M9 minimal salts 5X
5 g l^−1^ NaCl 10 g l^−1^ tryptone 5 g l^−1^ yeast extract	15 g l^−1^ KH_2_PO_4_ 2.5 g l^−1^ NaCl 33.9 g l^−1^ Na_2_HPO_4_ 15 g l^−1^ NH_4_Cl

Culture media, buffers and stock solutions were sterilized by autoclaving in a Priorclave EV150 top-loading autoclave at 121°C for 15 min. Solutions that could not be autoclaved were filter sterilized via 0.22 µm PES membrane disposable filter units from Merck Millipore Ltd. (Tullagreen, Ireland). All solutions were prepared using deionized water.

Optical density values were recorded at 550 nm wavelength in 1 ml aliquots in polystyrene semi-micro cuvettes from Greiner Bio-One (Kremsmünster, Austria) using a Camspec M107 spectrophotometer. Luminescence values were measured as relative light units in 1 ml aliquots (RLU ml^−1^) in polypropylene round-bottom luminometer cuvettes from Greiner Bio-One (Kremsmünster, Austria) using a Bio-Orbit Jade 1253 luminometer (Turku, Finland) at ambient temperature.

### GM bacterial strains and standard growth conditions

*Lux*-marked bacterial strains *P. putida* F1 Tn5 *lux*CDABE (soil isolate), *P. fluorescens* 8866 Tn5 *lux*CDABE (soil isolate) (Weitz et al. 2001), *P. putida* 12 708 Tn5 *lux*CDABE (freshwater isolate), and *Escherichia coli* HB101 pUCD607 (Rattray et al. 1990) were kindly provided by Prof. Graeme Paton and Dr. Lenka Mbadugha (University of Aberdeen).

All stock cultures were routinely grown on LB agar (Lennox) containing 50 µg ml^−1^ kanamycin at 25°C (see components in Table 1). For batch cultures, a single colony of cells from a LB plate (<1-week old) was used to inoculate 10 ml LB-Lennox Broth and grown overnight (12–14 h). A 1 ml aliquot of the overnight culture (OD_550nm_ ~ 2.0) was subsequently used to inoculate 250 ml conical flasks containing 100 ml LB Lennox Broth. The cultures were incubated at 25°C, at 200 rpm in an orbital shaking incubator. Aliquots from the batch cultures were sampled every 90 min for the measurement of optical density, luminescence, and colony forming units. All assays were carried out in triplicate and the standard error of the mean (SEM) calculated. The drop plate method was used to determine culturable cell count numbers (Hoben and Somasegaran 1982). Briefly, samples were serially diluted in quarter-strength Ringer’s solution, and triplicate 10 µl drops were pipetted onto an LB agar plate that was incubated at 25°C for 24 h until discrete single colonies could be counted (between 5 and 80 colonies). The number of CFU ml^−1^ was calculated from the mean values of the triplicate drops for the replicate flasks.

**Table 2:** Growth rate (µ) and generation time (g) of *lux*-marked bacterial strains under different growth conditions.

Bacterial strain	Media	PFAS compound	Concentration (mg l^−1^)	µ (h^−1^)	g (h)
*P. putida* F1 Tn5 *lux*CDABE	LB-Lennox	–	–	0.90 ± 0.04	0.77 ± 0.03
*P. putida* 12708 Tn5 *lux*CDABE	LB-Lennox	–	–	1.18 ± 0.04	0.59 ± 0.02
*P. fluorescens* 8866 Tn5 *lux*CDABE	LB-Lennox	–	–	1.48 ± 0.09	0.47 ± 0.03
*P. putida* F1 Tn5 *lux*CDABE	M9 + Glucose	PFOA	0	0.51 ± 0.01	1.36 ± 0.03
			0.1	0.60 ± 0.04	1.16 ± 0.08
			1	0.58 ± 0.03	1.20 ± 0.06
			10	0.53 ± 0.02	1.31 ± 0.05
		PFNA	0	0.50 ± 0.01	1.39 ± 0.03
			0.1	0.26 ± 0.02	2.73 ± 0.19
			1	0.28 ± 0.03	2.54 ± 0.31
			10	0.30 ± 0.03	2.36 ± 0.24
		PFHxS	0	0.57 ± 0.02	1.22 ± 0.04
			0.1	0.45 ± 0.00	1.53 ± 0.01
			1	0.41 ± 0.00	1.67 ± 0.00
			10	0.42 ± 0.00	1.65 ± 0.01
*P. putida* 12708 Tn5 *lux*CDABE	M9 + Glucose	PFOA	0	0.37 ± 0.01	1.87 ± 0.07
			0.1	0.55 ± 0.01	1.27 ± 0.03
			1	0.51 ± 0.05	1.41 ± 0.14
			10	0.68 ± 0.01	1.02 ± 0.02
		PFNA	0	0.41 ± 0.09	2.13 ± 0.64
			0.1	0.34 ± 0.01	2.02 ± 0.04
			1	0.21 ± 0.03	3.49 ± 0.49
			10	0.19 ± 0.01	3.67 ± 0.28
		PFHxS	0	0.39 ± 0.06	1.87 ± 0.25
			0.1	0.46 ± 0.05	1.57 ± 0.18
			1	0.39 ± 0.04	1.81 ± 0.19
			10	0.78 ± 0.07	0.91 ± 0.08
		PFOS	0	0.41 ± 0.04	1.72 ± 0.18
			0.1	0.52 ± 0.03	1.36 ± 0.08
			1	0.25 ± 0.05	3.11 ± 0.50
			10	0.29 ± 0.05	2.56 ± 0.36

Data represent average calculated values ± SEM of two or three batch culture replicates.

### Fluoride toxicity assays

*Lux*-marked bacterial biosensors *P. putida* F1 Tn5 *lux*CDABE, *P. fluorescens* 8866 Tn5 *lux*CDABE, *P. putida* 12 708 Tn5 *lux*CDABE, and *E. coli* HB101 pUCD607 were used to perform the bioluminescence-based bioassays. Bacterial cultures were grown in LB Broth (Lennox) as described in Section ‘GM bacterial strains and standard growth conditions’ for harvesting during late exponential phase, whereby optimal bioluminescence was observed. Cells were then washed by centrifuging 1 ml aliquots of batch cultures for 1 min at 7550 rpm. The supernatant was discarded and the pellets were resuspended in 1 ml of either 0.1 M KCl solution, or M9 1X media, following the washing procedure described by Paton et al. (1995). Unwashed cells were included as an additional condition. For each washing condition, the washed cell suspensions were pooled to obtain a homogeneous suspension of sufficient volume and used immediately for the bioassay. Both washed (0.1 M KCl or M9 1X media) and unwashed cells (LB) were subsequently exposed to different concentrations of inorganic fluoride (0.25–1000 mg l^−1^) aqueous solutions using a bioassay protocol previously described by Paton et al. (1995). Untreated cells (0 mg l^−1^) were used as a control. In brief, 100 µl of the cell suspension was mixed with 900 µl of the standard solutions; deionized water was used as a negative control. Samples were incubated at room temperature for 15 min before reading the light output. All bioassays were performed in triplicate for each washing condition.

### Growth inhibition assays

A single colony of cells from a LB plate (<1-week old) was used to inoculate 10 ml M9 Minimal Media 1X supplemented with 2 mM MgSO_4_ and 4 mg ml^−1^ glucose as a carbon source (Choi et al. 2008, García-Franco et al. 2023) and grown overnight (18–20 h). A 1 ml aliquot of the overnight culture (OD_550nm_ ~ 2.0) was subsequently used to inoculate 250 ml conical flasks containing 100 ml of M9 Minimal Media 1X supplemented with 4 mg ml^−1^ glucose as a carbon source as well as the pertinent PFAS compounds at various concentrations: 0, 0.1, 1, and 10 mg l^−1^ (Chiriac et al. 2023). The flasks were sealed with rubber butyl stoppers, and the cultures were incubated at 25°C, at 200 rpm in an orbital shaking incubator. Aliquots from the batch cultures were sampled every 90 min for the measurement of optical density, luminescence, and colony forming units. All assays were carried out in duplicate or triplicate and the SEM calculated.

### Data treatment and analysis

Bacterial growth rates (µ) were calculated from replicate CFU ml^−1^ measurements by fitting a linear regression to the natural logarithm-transformed CFU ml^−1^ values against time during the exponential phase of each replicate growth curve using Microsoft Excel (version 2603). Generation times were subsequently calculated as Ln(2)/µ.

Fluoride toxicity bioassays were analysed in R (version 4.6.0) using two-way analysis of variance (ANOVA) with fluoride concentration and cell washing condition (LB-Lennox, M9 or 0.1 M KCl) as fixed factors. Tukey-adjusted post hoc comparisons were used to compare cell washing conditions at each fluoride concentration. EC_50_ values, defined as the fluoride concentration causing a 50% reduction in bioluminescence, were determined using GraphPad Prism Software (version 11.0.0) by fitting a dose response curve with a variable slope using non-linear regression analysis.

Growth parameters were analysed in R (version 4.6.0) by one-way ANOVA for each strain and PFAS compound using concentration as the fixed factor. Significant effects were followed by Dunnett-adjusted comparisons against the untreated control.

The effect of PFAS toxicity on bioluminescence was analysed in R (version 4.6.0). For each PFAS compound and concentration, bioluminescence inhibition was calculated for each biological replicate relative to the untreated control using the equation:


\begin{eqnarray*}
\textit{Inhibition}\ \left( \% \right) = \ \frac{{\textit{Control}\ \textit{bioluminescence} - \textit{Treatment}\ \textit{bioluminescence}}}{{\textit{Control}\ \textit{bioluminescence}}} \times 100.
\end{eqnarray*}

For *P. putida* F1 Tn5 *lux*CDABE, which displayed a distinct peak in bioluminescence, inhibition was calculated at the peak timepoint at 6 h. For *P. putida* 12 708 Tn5 *lux*CDABE, which showed a broader bioluminescence maximum, inhibition was calculated using the mean bioluminescence across the defined peak-response window between 7.5 and 12 h. Replicate-level inhibition values were summarized as mean ± SEM for each PFAS compound and concentration. To assess concentration-dependent effects within each PFAS compound, one-way ANOVA was performed using percentage inhibition as the response variable and concentration as the fixed factor. Where significant overall effects were detected, Dunnett-adjusted post hoc comparisons were used to compare each concentration with the untreated control. To compare compound-specific responses at equivalent exposure concentrations, additional one-way ANOVA was performed separately for each concentration using percentage inhibition as the response variable and PFAS compound as the fixed factor. Tukey-adjusted post hoc comparisons were then used to assess pairwise differences between PFAS compounds within each concentration. Normality of ANOVA residuals was assessed using the Shapiro–Wilk test. Statistical significance was accepted at *P* < 0.05.

## Results

### Characterization of *lux*-modified *Pseudomonas* strains

GM bacterial sensors *P. putida* F1 Tn5 *lux*CDABE, *P. putida* 12 708 Tn5 *lux*CDABE and *P. fluorescens* 8866 Tn5 *lux*CDABE strains were grown in LB-Lennox Broth as described in Section ‘GM bacterial strains and standard growth conditions’ to characterize growth and bioluminescence parameters in nutrient rich conditions (Fig. 1). *Pseudomonas putida* F1 Tn5 *lux*CDABE exhibited optimal bioluminescence at 6 h (Fig. S1), corresponding to a late exponential growth phase. The peak in bioluminescence in the case of *P. putida* 12 708 Tn5 *lux*CDABE and *P. fluorescens* 8866 Tn5 *lux*CDABE occurred at 7.5 h. For all studied strains, the culturable cell counts correlated with the OD_550nm_ (Fig. S1), and the light output per cell started decreasing as the bacteria entered exponential growth phase without recovery (Fig. S2). This decline is expected for *lux*-marked reporters. During rapid cell division, luciferase may be diluted among daughter cells while reducing power and metabolic resources are redirected towards biomass production, limiting FMNH_2_ availability and therefore reducing bioluminescence (Meighen 1991, Park et al. 2013).

**Figure 1:**
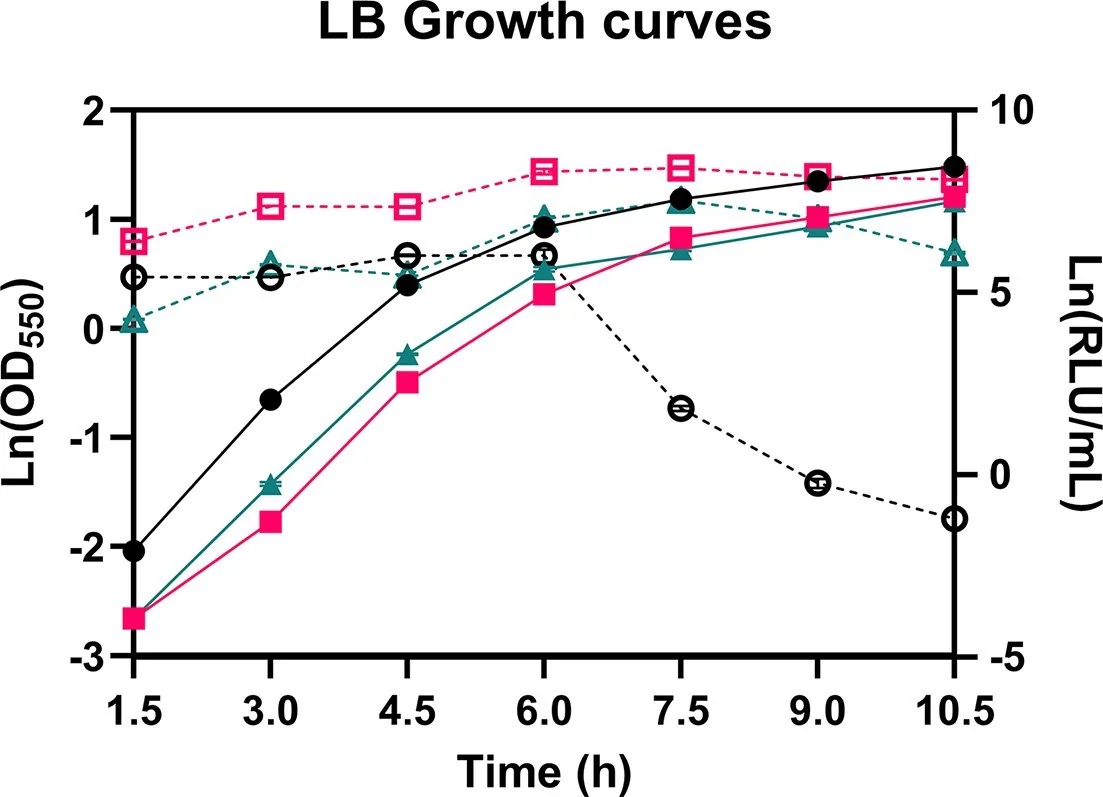
LB Broth Growth curves of *lux*-marked strains *P. putida* F1 Tn5 *lux*CDABE (•, ○), *P. putida* 12 708 Tn5 *lux*CDABE (▪, ⬜), and *P. fluorescens* 8866 Tn5 *lux*CDABE (▴, △). Ln(OD_550 nm_) measurements are displayed on the left *Y*-axis (•, ▪, ▴), while Ln(Bioluminescence; RLU/ml) is displayed on the right *Y*-axis (○, ⬜, △). Values show the mean of triplicate batch cultures ± SEM.

### Fluoride toxicity

Following the characterization of the *lux-*marked *Pseudomonas* strains, the effect of increasing concentrations of fluoride (0, 0.25, 0.5, 1.25, 2.5, 5, 12.5, 25, 50, 125, 250, 500, and 1000 mg l^−1^) was tested using a bioluminescence-based bioassay to understand the acute toxicity of this pollutant in the different strains. Fluoride sensitivity varied between unwashed (LB-Lennox) and washed cells (M9 or 0.1 M KCl) (Table S1). A significant interaction between cell preparation and fluoride concentration was observed for *P. putida* F1 Tn5 *lux*CDABE [*F*(24, 195) = 2.17, *P* = 0.002], indicating that washing altered fluoride sensitivity, particularly at higher fluoride concentrations. *Pseudomonas fluorescens* 8866 Tn5 *lux*CDABE showed a similar trend, although this was not significant overall (*P* = 0.088). Fluoride toxicity for *P. putida* 12 708 Tn5 *lux*CDABE [*F*(2, 195) = 3.24, *P* = 0.041] and *E. coli* HB101 pUCD607 [*F*(2, 195) = 5.99, *P* = 0.003] differed mainly according to cell preparation method, with unwashed cells generally displaying higher bioluminescence than washed cells across concentrations. Estimated marginal means and pairwise comparisons are provided in Tables S2–S9. The bioassays for *P. putida* F1 Tn5 *lux*CDABE and *P. putida* 12 708 Tn5 *lux*CDABE did not indicate toxicity. It was observed that both *P. putida* strains tolerated fluoride at high concentrations, as the bioluminescence was not affected after fluoride exposure. Meanwhile, *P. fluorescens* 8866 Tn5 *lux*CDABE exhibited signs of fluoride toxicity after 100 mg l^−1^, with a calculated best-fit EC_50_ value of ~281.7 mg l^−1^ fluoride (Fig. 2a). Bioluminescent *E. coli* HB101 pUCD607 was also tested for fluoride toxicity using the same bioassay for comparison. The toxic effects of fluoride on *E. coli* HB101 pUCD607 were milder than in the case of *P. fluorescens* 8866 Tn5 *lux*CDABE, with an EC_50_ of 1153 mg l^−1^ fluoride (Fig. 2b). Nonetheless, all strains including the *E. coli* comparison tolerated concentrations of fluoride up to 100 mg l^−1^.

**Figure 2:**
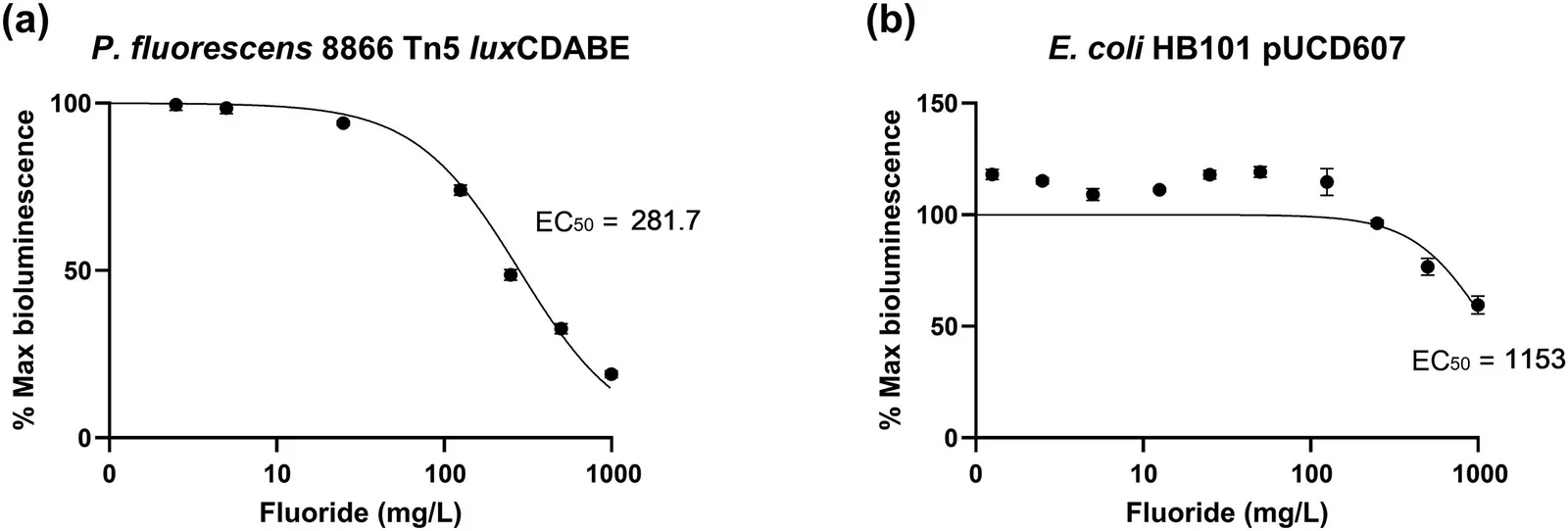
Dose-response curves for the fluoride toxicity effects on the bioluminescence of *P. fluorescens* 8866 Tn5 *lux*CDABE (a) and *E. coli* HB101 pUCD607 (b) cells washed and resuspended in 0.1 M KCl. Data shown are the mean values of triplicate bioassay samples ± SEM.

### PFAS toxicity effects in bioluminescent *Pseudomonas* strains

Bioluminescent *P. putida* strains shown to be tolerant to fluoride were grown in M9 minimal media supplemented with glucose (4 mg ml^−1^) in the presence of increasing concentrations of various PFAS compounds. Figures 3–4 illustrate the effects of PFOA, PFNA and PFHxS (0, 0.1, 1, and 10 mg l^−1^) on the growth and bioluminescence responses of *P. putida* F1 Tn5 *lux*CDABE and *P. putida* 12 708 Tn5 *lux*CDABE, respectively. Growth was assessed by CFU ml^−1^ (Figs. 3–4) and OD_550nm_ (Fig. S3). Quantitative growth parameters are shown in Table 2. PFOA exposure did not affect the growth of *P. putida* F1 Tn5 *lux*CDABE, whereas PFNA (*P* = 0.001) and PFHxS (*P* = 0.003) significantly reduced the growth of this strain relative to the control across all tested concentrations (Tables S10–S12). Linear mixed-effects models of OD_550nm_ and CFU ml^−1^ trajectories supported these trends, although trajectory-based results were interpreted cautiously due to singular fits and residual deviations in some models. In terms of bioluminescence, all tested PFAS compounds affected the light output emission of *P. putida* F1 Tn5 *lux*CDABE (Fig. 3b, d, f). PFOA and PFNA significantly inhibited the strain’s bioluminescence during the late exponential phase at 6 h (Tables S13–S15). PFOA at 10 mg l^−1^ reduced the bioluminescence of the *lux-*marked bacterial strain by 38% (Fig. 5a). Similarly, 10 mg l^−1^ of PFNA caused a light output reduction of 42.1%, although this was not statistically significant. Significant reductions in peak bioluminescence were caused by PFNA at 0.1 mg l^−1^ (*P* = 0.012) and 1 mg l^−1^ (*P* = 0.032) (Table S14). Not shown on the graphs in Fig. 5, 100 mg l^−1^ of PFNA exhibited a 66.3% reduction in maximum bioluminescence, and 30 mg l^−1^ of PFHxS caused a 40.1% light output reduction. Comparatively, the growth of *P. putida* 12 708 Tn5 *lux*CDABE was not negatively affected by the presence of PFAS compounds in the growth media between 0.1 and 10 mg l^−1^. However, growth rate analysis showed an increased growth rate relative to the control for PFOA at 0.1 and 10 mg l^−1^, and for PFHxS at 10 mg l^−1^ (Table S11). Bioluminescence responses of *P. putida* 12 708 Tn5 *lux*CDABE differed from those of *P. putida* F1 Tn5 *lux*CDABE (Tables S16–S18). No inhibition was observed for PFOA and PFHxS (Fig. 4b, f). The sustained bioluminescence of *P. putida* 12 708 Tn5 *lux*CDABE reflects the lower cell densities attained in M9 1X minimal medium compared with LB, which could limit dilution of the luciferase pool and metabolic competition for reducing equivalents, sustaining light output in absence of acute toxicity (Falls et al. 2014). In the case of PFOA, concentrations 0.1 and 1 mg l^−1^ caused a non-significant increase in light output emission over the control in late exponential growth phase (Fig. 5b). Significant inhibition relative to the control was found at 100 mg l^−1^ for PFOA (*P* = 0.008) and PFHxS (*P* = 0.042) (Table S17). PFNA and PFOS did not show any significant concentration-dependent responses, even though visible reductions in light output were observed, including a 39.9% inhibition at 1 mg l^−1^ PFNA, and an 88% reduction at 10 mg l^−1^ PFOS (Fig. S4c).

**Figure 3:**
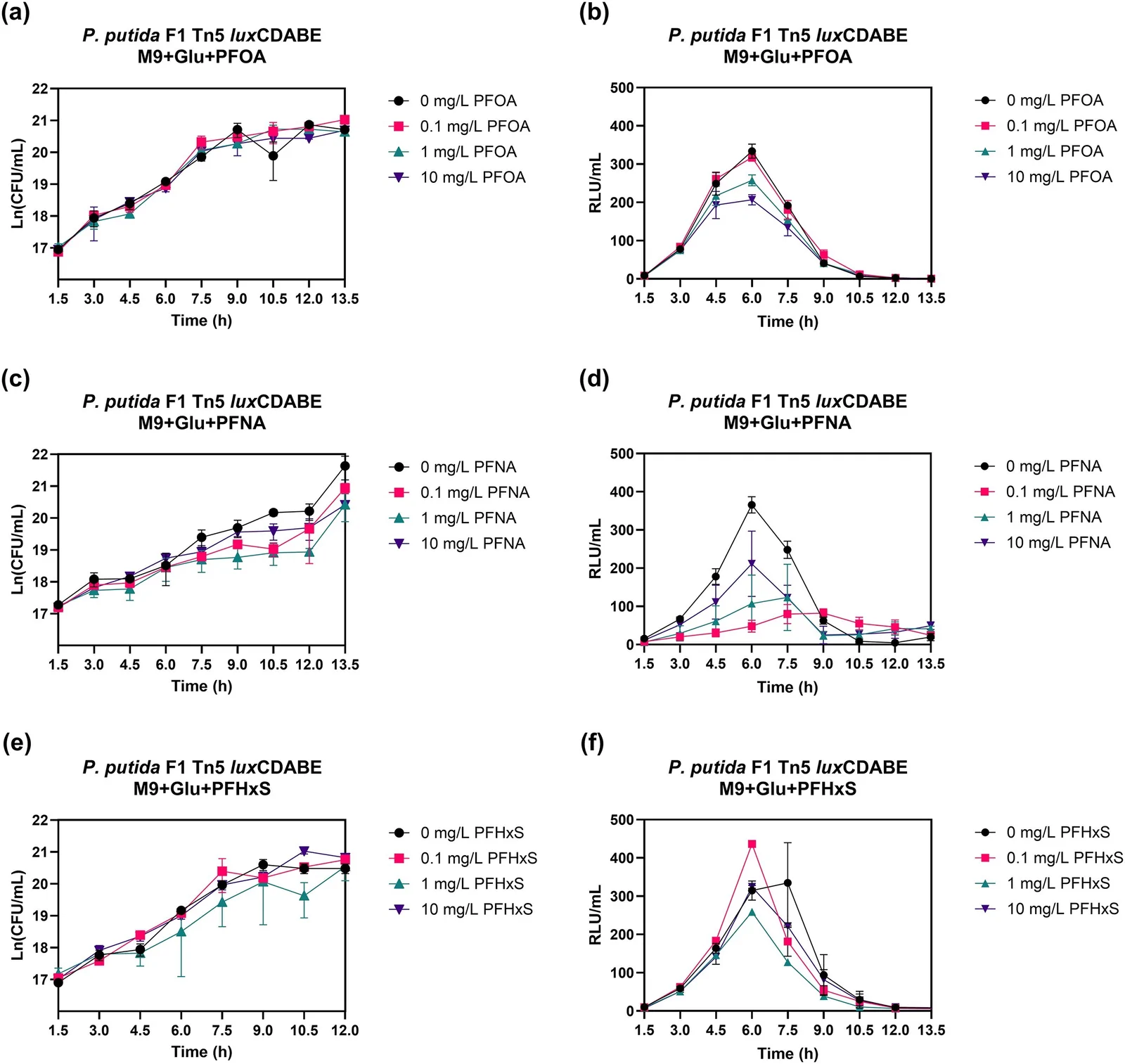
Effect of PFAS on the growth (a, c, e) and bioluminescence (b, d, f) of *P. putida* F1 Tn5 *lux*CDABE in M9 minimal media supplemented with 0.4% glucose as a source of carbon: PFOA (a–b), PFNA (c–d), and PFHxS (e–f). Values shown represent the mean of duplicate batch cultures ± SEM (a–b, e–f), and triplicate batch cultures ± SEM (c, d).

**Figure 4:**
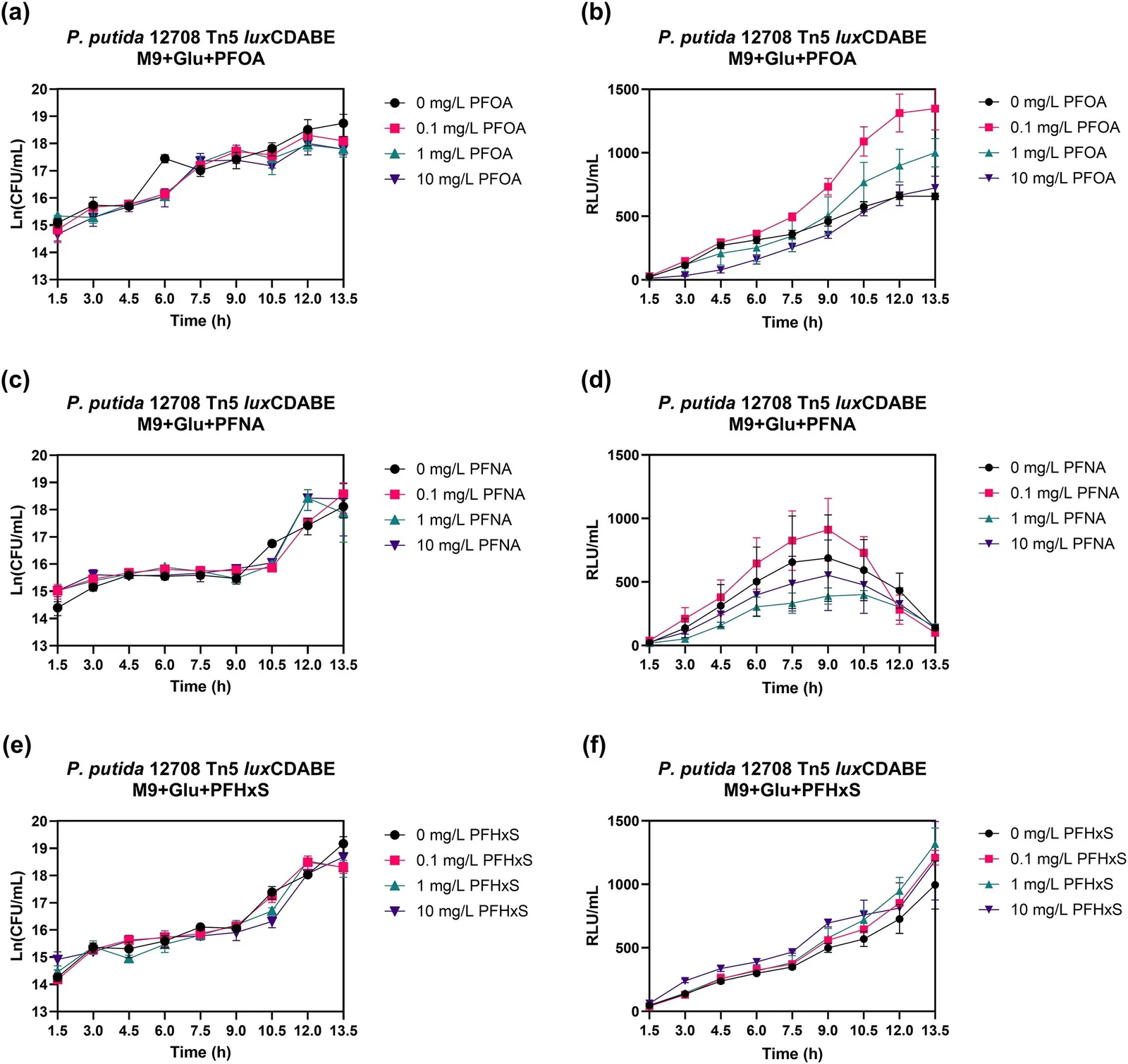
Effect of PFAS on the growth (a, c, e) and bioluminescence (b, d, f) of *P. putida* 12 708 Tn5 *lux*CDABE in M9 minimal media supplemented with 0.4% glucose as a source of carbon: PFOA (a–b), PFNA (c–d), and PFHxS (e–f). Values show the mean of triplicate batch cultures ± SEM.

**Figure 5:**
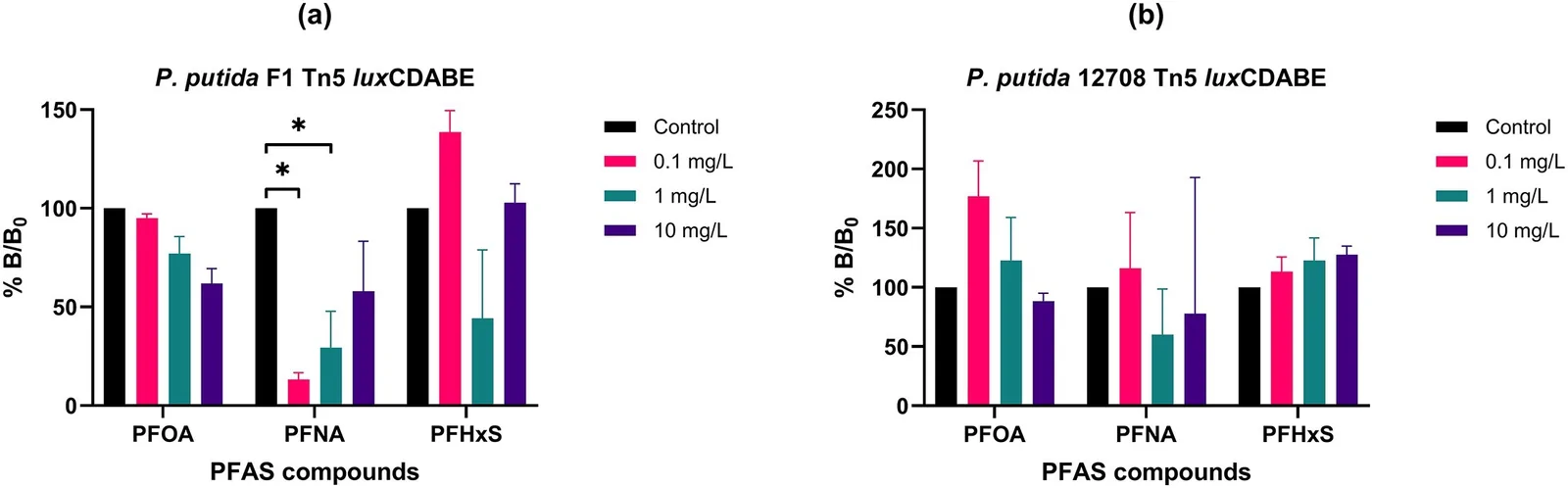
Effect of PFAS exposure on peak bioluminescence in *P. putida* strains expressed as relative luminescence (B/B_0_, %). Peak bioluminescence inhibition responses of (a) *P. putida* F1 Tn5 *lux*CDABE at 6 h and (b) *P. putida* 12 708 Tn5 *lux*CDABE across the peak window between 7.5 and 12 h, following exposure to PFOA, PFNA and PFHxS at 0.1, 1, and 10 mg l^−1^. Bioluminescence is presented as B/B_0_ (%), where B represents bioluminescence under PFAS exposure and B_0_ represents the untreated control (0 mg l^−1^), normalized to 100%. Values are shown as mean ± SEM of two or three biological replicates. Statistical differences between concentrations and the control were determined for each PFAS compound by one-way ANOVA followed by Dunnett’s multiple comparison test (*P* < 0.05).

Comparisons between PFAS compounds at equivalent concentrations highlighted the sensitivity of *P. putida* F1 Tn5 *lux*CDABE. At 0.1 mg l^−1^, this strain showed distinct compound-specific responses, with PFNA causing the strongest inhibition of peak bioluminescence, followed by PFHxS, while PFOA produced the lowest response (*P* < 0.05) (Tables S19–S21). No significant differences between compounds were detected at 1 or 10 mg l^−1^. In contrast, *P. putida* 12 708 Tn5 *lux*CDABE did not show any clear compound-specific differences (Tables S22–S24).

Table 2 summarizes quantitative growth parameters of the studied *lux*-modified *Pseudomonas* strains under various growth conditions and PFAS exposures. Baseline growth parameters were determined in LB-Lennox medium in the absence of PFAS. Growth and toxicity responses to PFAS compounds were assessed in M9 minimal medium supplemented with glucose (4 mg ml^−1^) as a carbon source in the presence of PFOA, PFNA, PFHxS, and PFOS across a concentration range of 0–10 mg l^−1^.

Growth kinetics varied by species, growth medium, and PFAS exposure (Table 2). Under nutrient-rich LB-Lennox conditions, *P. fluorescens* 8866 Tn5 *lux*CDABE exhibited the highest growth rate (µ = 1.48 ± 0.09 h^−1^), followed by *P. putida* 12 708 Tn5 *lux*CDABE (µ = 1.18 ± 0.04 h^−1^), and *P. putida* F1 Tn5 *lux*CDABE (µ = 0.90 ± 0.04 h^−1^). As anticipated, growth in M9 minimal medium resulted in reduced growth rates and increased generation times across all strains. For *P. putida* F1 Tn5 *lux*CDABE, exposure to PFOA resulted in only minor changes in growth parameters across the tested concentration range (µ = 0.51–0.60 h^−1^), whereas PFNA and PFHxS significantly caused a reduction in growth rate relative to the control.

In contrast, *P. putida* 12 708 Tn5 *lux*CDABE displayed increased growth rates in response to PFOA and PFHxS at selected concentrations, with the highest value observed for PFHxS at 10 mg l^−1^ (µ = 0.78 ± 0.07 h^−1^). PFNA did not significantly alter the growth of this strain, while PFOS showed an overall concentration effect without significant pairwise differences after correction.

It must be noted that the growth curves for this strain grown in minimal medium exhibited non-classical patterns characterized by an initial increase in optical density and CFU counts, followed by a transient lag phase and a subsequent second phase of exponential growth. This effect was more pronounced under PFAS exposure, particularly at higher concentrations (10 mg l^−1^), where an extended lag phase preceded exponential growth. These observations indicate that PFAS exposure primarily affected the timing of growth rather than the capacity for exponential growth itself. Accordingly, calculated growth rates (µ) reflect only the later exponential phase and may not capture early growth inhibition. The prolonged lag phases observed at increasing PFAS concentrations suggest that cells undergo a period of physiological adaptation prior to resuming growth, consistent with a stress response rather than acute toxicity. The lack of significant pH change in the media also suggests that these strains are navigating this membrane stress through tolerance rather than mineralization.

Overall, PFAS exposure elicited compound- and concentration-dependent effects on *P. putida* growth kinetics, with both inhibitory and stimulatory responses observed depending on strain and conditions. In parallel, bioluminescence responses were also altered by PFAS exposure and exhibited greater sensitivity than traditional growth metrics, indicating that metabolic disruption may occur even where changes in growth are less apparent.

## Discussion

PFAS are characterized by highly stable C–F bonds, which underpin their environmental persistence and their resistance to degradation (Evich et al. 2022). Although PFAS toxicity stems from the intact molecular compounds rather than free fluoride release (Evich et al. 2022, Basu 2024, Stockbridge and Wackett 2024), assessing bacterial responses to fluoride provides useful context for understanding tolerance to organic fluorinated compounds and can help distinguish PFAS-associated stress from fluoride-specific effects. Fluoride is not uniformly lethal to bacteria, and its apparent toxicity depends strongly on concentration, exposure time, pH, and the presence of fluoride efflux transporters such as CrcB or Fluc (Ji et al. 2014, Calero et al. 2020, Calero et al. 2022, Smorada et al. 2024). Ma et al. (2014) reported that fluoride stimulated growth in *E. coli* at low concentrations but inhibited growth above 10 mM NaF (~190 mg l^−1^ F^−^), whereas Breaker (2012) reported that *E. coli* cannot survive long exposure of fluoride close to 250 mM (4750 mg l^−1^). In this study, *E. coli* HB101 pUCD607 only exhibited a reduction in bioluminescence at a fluoride concentration over 500 mg l^−1^. Calero et al. (2022) found that *P. putida* KT2440 could withstand up to 50–75 mM NaF (>900 mg l^−1^ F^−^) in M9 minimal medium, and up to 125 mM NaF (>2000 mg l^−1^ F^−^) in LB. These results support the ones found in this study, where both GM *P. putida* F1 Tn5 *lux*CDABE and *P. putida* 12 708 Tn5 *lux*CDABE exhibited substantial tolerance to fluoride up to 1000 mg l^−1^ with no change in bioluminescence. Washing the cells with a 0.1 M KCl solution produced the most consistent and sensitive bioluminescence responses, in line with Weitz et al. (2001). Fluoride toxicity can be masked when ions are chelated or buffered by complex growth media components (Stockbridge and Wackett 2024), therefore washing the cells likely improved signal detection by removing these interferents. Together, these findings demonstrate that fluorinated compounds are not universally toxic to these organisms, while further consolidating the use of *lux*-based systems in ecotoxicology as they are capable of detecting toxic effects.

PFAS exposure elicited more complex responses that were strongly dependent on compound structure, concentration, and bacterial strain. Bioluminescent responses across both *P. putida* strains were more sensitive to PFAS exposure than conventional growth measurements. In several cases, reductions in light output were observed in the absence of measurable changes in optical density or viable culturable cell counts (Marques et al. 2005), indicating that PFAS-induced metabolic disruption can occur prior to detectable growth inhibition (Patil et al. 2025). While bioluminescence broadly followed growth trends, discrepancies between luminescence and OD or CFU measurements highlight that these metrics capture different aspects of cellular physiology and are more closely linked to bacterial metabolism (Wang et al. 2020). In addition, OD measurements may be influenced by EPS production, common in *Pseudomonas* soil isolates as environmental adaptation by means of protection from contaminated sites (Weimer et al. 2020, Bedoya Vélez et al. 2021, Kaur and Dey 2023), even though defined M9 Minimal Medium reduces biofilm formation in *P. putida (* *Puhm et al. 2022*). PFAS compounds have been observed to trigger stress responses in *Pseudomonas* via the production of exopolysaccharides (LaFond et al. 2023). Moreover, PFAS compounds at high concentrations can start losing water solubility as they self-associate, adsorb to interfaces, or form aggregates (Kancharla et al. 2022, Creton et al. 2024, Klevan et al. 2024, Sobolewski et al. 2024), which could further affect the OD readings. Due to these reasons, growth parameters were calculated from cell counts as opposed to OD values. All of these considerations support the use of bioluminescence as a more sensitive indicator of cellular stress. Observed variability between replicates likely reflects the high sensitivity of the bioluminescent response to subtle physiological changes rather than experimental inconsistency (Kong et al. 2022, Patil et al. 2025). This variability may also reflect bistability, a phenomenon in which genetically identical bacterial populations partition into distinct phenotypic subpopulations in response to stress, with individual cells switching between alternative metabolic states in a threshold-dependent manner (Dubnau and Losick 2006). Because the measured light output integrates the metabolic state of the entire culture, small differences between replicate cultures in the fraction of cells occupying each state can translate into measurable differences in bioluminescence. Population-level bistability has recently been demonstrated in *Pseudomonas* quorum-sensing systems (Estell and Schuster 2025), supporting the plausibility of such population-level heterogeneity in the responses observed here.

The most consequential finding in this study is that bioluminescence was reduced at PFAS concentrations where OD_550nm_ and CFU ml^−1^ remained essentially unchanged. While biomass remained stable at PFAS concentrations up to 10 mg l^−1^, light output was significantly inhibited. Bacterial *lux* reporters are inherently sensitive to perturbations in cellular energy status and metabolic flux because the luciferase enzyme requires a reduced flavin mononucleotide (FMNH_2_) tightly coupled to oxidative metabolism (Park et al. 2013). When a chemical stressor interferes with these processes at a sub-lethal level, light output falls before any measurable loss of growth. The observed reduction in light output without a corresponding drop in OD suggests that PFAS are acting as metabolic disruptors. This aligns with Panella et al. (2025) who found that PFAS can destabilize the lipid bilayer, increasing membrane permeability. This makes bioluminescence-based assays more sensitive than conventional growth inhibition tests for surfactants such as PFAS.

The observed variability between compounds is likely related to differences in PFAS structure, including chain length and functional group. PFNA (C_9_HF_17_O_2_), with a 9-carbon backbone, was the strongest inhibitor across both bacterial strains, whereas PFHxS (C_6_F_13_SO_3_H), the shortest chain compound tested, with a 6-carbon backbone and a sulfonic acid group, caused the lowest reduction in bioluminescence at the same concentration. This clear structure–activity relationship regarding toxicity is consistent with studies showing that longer carbon chains confer greater lipophilicity and stronger interactions with bacterial membranes (Butzen et al. 2020, Dai et al. 2023, Fosella et al. 2025). The extreme inhibition caused by PFOS in *P. putida* 12 708 Tn5 *lux*CDABE (85.6% light reduction at 10 mg l^−1^) (Fig. S4) compared to its carboxylate counterpart, PFOA, highlights the higher toxicity of the sulfonate head group (Liu et al. 2016). Interestingly, PFOA caused an increase in light emission relative to the control in *P. putida* 12 708 Tn5 *lux*CDABE, following the hormesis phenomenon, which occurs when sub-toxic levels of stressors trigger a compensatory adaptive response (Si et al. 2024).

The concentration range tested (0–10 mg l^−1^) is several orders of magnitude above typical environmental PFAS contamination levels (Abunada et al. 2020) and established regulatory limits for PFAS in drinking water (0.1 µg l^−1^) (European Parliament and Council of the European Union 2020, Drinking Water Inspectorate 2025) and food systems (0.1–50 µg kg^−1^) (European Commission and Directorate-General for Health and Food Safety 2023). However, the high tolerance of these *lux*-marked *Pseudomonas* biosensors suggest that these reporters could serve as early-warning sensors for PFAS contamination at much lower levels if optimized further. The survival of these strains at high concentrations without growth impairment positions them as positive indicators for its viability in heavy polluted sites (Shahsavari et al. 2020) as well as robust tools for future engineering (Wackett 2024). Even though the pervasiveness of the C-F bond in PFAS molecules makes complete defluorination extremely challenging (Wackett 2025), microbial biotransformation of some of these compounds has been studied. *Pseudomonas* species have been described as capable of PFAS biodegradation (Kwon et al. 2014, Yi et al. 2019, Chetverikov et al. 2023, Chiriac et al. 2023), although the mechanisms of degradation remain poorly understood, making tolerance studies a necessary prerequisite for any biosensing or bioremediation application. In this study, *Pseudomonas putida* strains were grown on glucose in the presence of PFAS, which may have supported co-metabolic processes. Previous studies suggested that PFAS are not ideally utilized as primary carbon sources, but may undergo partial transformation in the presence of readily metabolized substrates (LaFond et al. 2023, Smorada et al. 2024).

While the concentrations tested in this study exceed those typically reported in environmental and regulatory contexts, the observed sensitivity of bioluminescence responses highlights the potential of these systems for ecotoxicological screening. Further optimization would be required to achieve detection limits relevant to current regulatory thresholds. It must also be noted that some of the assays in this study were performed in duplicate rather than triplicate, and additional replicates would be required in order to confirm the statistical conclusions with full confidence. Although the low variability between replicate measurements supports the reproducibility of the reported trends, the findings should be regarded as a proof-of-principle for the use of *lux*-marked *Pseudomonas* biosensors in PFAS toxicity assessment. Nonetheless, these findings demonstrate the utility of whole-cell bioluminescent biosensors as rapid and sensitive tools for detecting sublethal toxicity. Overall, this study demonstrates that *lux*-marked *Pseudomonas* biosensors can provide a sensitive means of detecting PFAS-induced metabolic stress, revealing effects that are not captured by conventional growth assays and highlighting the importance of both strain selection and compound-specific properties in ecotoxicological assessment. More broadly, the observed microbial responses suggest that interactions between PFAS and environmental microorganisms may be more dynamic than previously assumed, challenging the perception of these compounds as biologically inert ‘forever chemicals’. Future work should also assess microbial responses to environmentally relevant complex PFAS mixtures, where cumulative, antagonistic, or competitive interactions between compounds may influence toxicity differently from individual compound exposure.

## Supplementary Material

fnag135_Supplemental_File

## Data Availability

The data underlying this article will be shared on reasonable request to the corresponding author.
