# Supplementary material for: Assessment of PFAS toxicity using lux-marked bioluminescent Pseudomonas biosensors
Source: FEMS Microbiol Lett. 2026 Oct 30;373:fnag135. doi: 10.1093/femsle/fnag135 (PMC13762462; doi:10.1093/femsle/fnag135)
Supplement: Supplementary file 1 [file fnag135_supplemental_file.docx]

**Supplementary Information**

**Figure S1_Supplementary Data**

Fig. S1: (a) Cell counts of growth curves of *lux*-marked strains *P. putida* F1 Tn5 *lux*CDABE (●, ○), *P. putida* 12708 Tn5 *lux*CDABE (■, □) and *P. fluorescens* 8866 Tn5 *lux*CDABE (▲, △) in LB-Lennox Broth. Ln(OD_550 nm_) measurements are displayed on the left Y-axis (●, ■, ▲), while Ln(CFU/ml) values are displayed on the right Y-axis (○, □, △). (b) Bioluminescence of *lux*-marked strains *P. putida* F1 Tn5 *lux*CDABE (●) (displayed on right Y-axis), *P. putida* 12708 Tn5 *lux*CDABE (■) (left Y-axis) and *P. fluorescens* 8866 Tn5 *lux*CDABE (▲) (left Y-axis) in LB-Lennox Broth. Data shown is the mean of triplicate batch cultures ± SEM.

**Figure S2_Supplementary Data**

Fig. S2: Light output per cell for *P. putida* F1 Tn5 *lux*CDABE (●), *P. fluorescens* 8866 Tn5 *lux*CDABE (▲) (a) and *P. putida* 12708 Tn5 *lux*CDABE (■) (b) grown in LB-Lennox Broth. Data shown is the mean of triplicate batch cultures ± SEM.

**Figure S3_Supplementary Data**

Fig. S3: OD_550 nm_ values for the growth of *P. putida* F1 Tn5 *lux*CDABE (a, c, e) and *P. putida* 12708 Tn5 *lux*CDABE (b, d, f) in M9 minimal media supplemented with 0.4% glucose as a source of carbon under the presence of different PFAS concentrations: PFOA (a-b), PFNA (c-d), and PFHxS (e-f). Values represent the mean of duplicate (a, e) and triplicate (b-d, f) batch cultures ± SEM.

**Figure S4_Supplementary Data**

Fig. S4: Effect of PFOS at various concentrations on the growth (a), bioluminescence (b) and optimal bioluminescence (c) of *P. putida* 12708 Tn5 *lux*CDABE grown in M9 minimal media supplemented with 0.4% glucose as a source of carbon. (a) Solid symbols represent Ln(OD_550 nm_) values plotted on the left Y-axis, and hollow symbols correspond to Ln(CFU mL^-1^) counts plotted on the right Y-axis. Data represent the values of three replicates ± SEM.

**Table S1**

Table S1: Two-way ANOVA (Type III sums of squares) of fluoride concentration and cell preparation condition (LB-Lennox, M9 or 0.1 M KCl) on bioluminescence in *lux*-marked bacterial strains *P. putida* F1 Tn5 *lux*CDABE, *P. putida* 12708 Tn5 *lux*CDABE, *P. fluorescens* 8866 Tn5 *lux*CDABE, and *E. coli* HB101 pUCD607. Significant effects (*p* < 0.05) are shown in bold.

| Strain | Source | Sum Sq | Df | F value | Pr(>F) |
| --- | --- | --- | --- | --- | --- |
| *P. putida* F1 Tn5 *lux*CDABE | Fluoride concentration | 8508 | 12 | 1.60 | 0.093 |
|  | Cell preparation (washed vs unwashed) | 1662 | 2 | 1.88 | 0.155 |
|  | Interaction | 22979 | 24 | 2.17 | **0.002** |
|  | Residuals | 86154 | 195 | - | - |
| *P. putida* 12708 Tn5 *lux*CDABE | Fluoride concentration | 298797 | 12 | 0.14 | 0.9998 |
|  | Cell preparation (washed vs unwashed) | 1183413 | 2 | 3.24 | **0.041** |
|  | Interaction | 1477123 | 24 | 0.34 | 0.999 |
|  | Residuals | 35564618 | 195 | - | - |
| *P. fluorescens* 8866 Tn5 *lux*CDABE | Fluoride concentration | 75869 | 12 | 1.02 | 0.434 |
|  | Cell preparation (washed vs unwashed) | 11926 | 2 | 0.96 | 0.385 |
|  | Interaction | 216313 | 24 | 1.45 | 0.088 |
|  | Residuals | 1211778 | 195 | - | - |
| *E. coli* HB101 pUCD607 | Fluoride concentration | 5705 | 12 | 0.36 | 0.974 |
|  | Cell preparation (washed vs unwashed) | 15619 | 2 | 5.99 | **0.003** |
|  | Interaction | 25038 | 24 | 0.80 | 0.734 |
|  | Residuals | 254259 | 195 | - | - |

**Table S2**

Table S2: Estimated marginal means (emmeans) of bioluminescence responses (B/B_0_, %) across fluoride concentrations and cell preparation conditions for *P. putida* F1 Tn5 *lux*CDABE.

| Strain: *P. putida* F1 Tn5 *lux*CDABE | | | | | | |
| --- | --- | --- | --- | --- | --- | --- |
| Condition | **Fluoride (mg L^-1^)** | **emmean** | **SE** | **df** | **lower.CL** | **upper.CL** |
| LB | 0 | 98.31 | 8.58 | 195 | 81.38 | 115.23 |
| M9 | 0 | 82.49 | 8.58 | 195 | 65.56 | 99.41 |
| KCl | 0 | 75.30 | 8.58 | 195 | 58.38 | 92.23 |
| LB | 0.25 | 98.00 | 8.58 | 195 | 81.08 | 114.93 |
| M9 | 0.25 | 82.25 | 8.58 | 195 | 65.32 | 99.17 |
| KCl | 0.25 | 79.81 | 8.58 | 195 | 62.89 | 96.74 |
| LB | 0.5 | 101.34 | 8.58 | 195 | 84.41 | 118.26 |
| M9 | 0.5 | 81.58 | 8.58 | 195 | 64.66 | 98.50 |
| KCl | 0.5 | 77.10 | 8.58 | 195 | 60.17 | 94.02 |
| LB | 1.25 | 96.70 | 8.58 | 195 | 79.78 | 113.63 |
| M9 | 1.25 | 79.24 | 8.58 | 195 | 62.32 | 96.16 |
| KCl | 1.25 | 76.13 | 8.58 | 195 | 59.20 | 93.05 |
| LB | 2.5 | 100.37 | 8.58 | 195 | 83.45 | 117.29 |
| M9 | 2.5 | 77.36 | 8.58 | 195 | 60.44 | 94.29 |
| KCl | 2.5 | 73.70 | 8.58 | 195 | 56.78 | 90.63 |
| LB | 5 | 100.29 | 8.58 | 195 | 83.36 | 117.21 |
| M9 | 5 | 78.36 | 8.58 | 195 | 61.44 | 95.29 |
| KCl | 5 | 73.17 | 8.58 | 195 | 56.25 | 90.10 |
| LB | 12.5 | 98.47 | 8.58 | 195 | 81.55 | 115.39 |
| M9 | 12.5 | 72.06 | 8.58 | 195 | 55.14 | 88.99 |
| KCl | 12.5 | 74.50 | 8.58 | 195 | 57.58 | 91.42 |
| LB | 25 | 100.62 | 8.58 | 195 | 83.70 | 117.55 |
| M9 | 25 | 72.82 | 8.58 | 195 | 55.90 | 89.75 |
| KCl | 25 | 72.78 | 8.58 | 195 | 55.86 | 89.71 |
| LB | 50 | 100.22 | 8.58 | 195 | 83.30 | 117.15 |
| M9 | 50 | 72.99 | 8.58 | 195 | 56.06 | 89.91 |
| KCl | 50 | 76.85 | 8.58 | 195 | 59.92 | 93.77 |
| LB | 125 | 99.74 | 8.58 | 195 | 82.82 | 116.67 |
| M9 | 125 | 73.30 | 8.58 | 195 | 56.38 | 90.23 |
| KCl | 125 | 83.75 | 8.58 | 195 | 66.83 | 100.67 |
| LB | 250 | 107.68 | 8.58 | 195 | 90.75 | 124.60 |
| M9 | 250 | 76.49 | 8.58 | 195 | 59.57 | 93.42 |
| KCl | 250 | 100.40 | 8.58 | 195 | 83.47 | 117.32 |
| LB | 500 | 109.97 | 8.58 | 195 | 93.04 | 126.89 |
| M9 | 500 | 86.37 | 8.58 | 195 | 69.45 | 103.30 |
| KCl | 500 | 98.40 | 8.58 | 195 | 81.48 | 115.33 |
| LB | 1000 | 137.76 | 8.58 | 195 | 120.83 | 154.68 |
| M9 | 1000 | 153.17 | 8.58 | 195 | 136.24 | 170.09 |
| KCl | 1000 | 71.18 | 8.58 | 195 | 54.26 | 88.10 |

**Table S3**

Table S3: Estimated marginal means (emmeans) of bioluminescence responses (B/B_0_, %) across fluoride concentrations and cell preparation conditions for *P. putida* 12708 Tn5 *lux*CDABE.

| Strain: *P. putida* 12708 Tn5 *lux*CDABE | | | | | | |
| --- | --- | --- | --- | --- | --- | --- |
| Condition | **Fluoride (mg L^-1^)** | **emmean** | **SE** | **df** | **lower.CL** | **upper.CL** |
| LB | 0 | 651.83 | 174.35 | 195 | 307.98 | 995.68 |
| M9 | 0 | 164.22 | 174.35 | 195 | -179.63 | 508.07 |
| KCl | 0 | 65.20 | 174.35 | 195 | -278.64 | 409.05 |
| LB | 0.25 | 655.64 | 174.35 | 195 | 311.79 | 999.49 |
| M9 | 0.25 | 162.16 | 174.35 | 195 | -181.68 | 506.01 |
| KCl | 0.25 | 65.10 | 174.35 | 195 | -278.75 | 408.95 |
| LB | 0.5 | 622.00 | 174.35 | 195 | 278.15 | 965.85 |
| M9 | 0.5 | 159.94 | 174.35 | 195 | -183.91 | 503.79 |
| KCl | 0.5 | 55.47 | 174.35 | 195 | -288.38 | 399.32 |
| LB | 1.25 | 643.36 | 174.35 | 195 | 299.51 | 987.21 |
| M9 | 1.25 | 155.33 | 174.35 | 195 | -188.52 | 499.18 |
| KCl | 1.25 | 71.76 | 174.35 | 195 | -272.08 | 415.61 |
| LB | 2.5 | 664.80 | 174.35 | 195 | 320.95 | 1008.65 |
| M9 | 2.5 | 157.90 | 174.35 | 195 | -185.95 | 501.75 |
| KCl | 2.5 | 54.25 | 174.35 | 195 | -289.60 | 398.10 |
| LB | 5 | 650.74 | 174.35 | 195 | 306.89 | 994.59 |
| M9 | 5 | 147.27 | 174.35 | 195 | -196.58 | 491.12 |
| KCl | 5 | 56.21 | 174.35 | 195 | -287.64 | 400.06 |
| LB | 12.5 | 647.50 | 174.35 | 195 | 303.66 | 991.35 |
| M9 | 12.5 | 150.73 | 174.35 | 195 | -193.12 | 494.58 |
| KCl | 12.5 | 47.52 | 174.35 | 195 | -296.33 | 391.37 |
| LB | 25 | 676.59 | 174.35 | 195 | 332.74 | 1020.44 |
| M9 | 25 | 150.06 | 174.35 | 195 | -193.79 | 493.91 |
| KCl | 25 | 57.96 | 174.35 | 195 | -285.89 | 401.81 |
| LB | 50 | 680.17 | 174.35 | 195 | 336.32 | 1024.02 |
| M9 | 50 | 105.56 | 174.35 | 195 | -238.29 | 449.41 |
| KCl | 50 | 65.80 | 174.35 | 195 | -278.05 | 409.65 |
| LB | 125 | 738.66 | 174.35 | 195 | 394.82 | 1082.51 |
| M9 | 125 | 160.48 | 174.35 | 195 | -183.37 | 504.33 |
| KCl | 125 | 154.16 | 174.35 | 195 | -189.69 | 498.01 |
| LB | 250 | 776.58 | 174.35 | 195 | 432.73 | 1120.43 |
| M9 | 250 | 185.60 | 174.35 | 195 | -158.25 | 529.45 |
| KCl | 250 | 318.90 | 174.35 | 195 | -24.95 | 662.75 |
| LB | 500 | 852.02 | 174.35 | 195 | 508.17 | 1195.87 |
| M9 | 500 | 257.68 | 174.35 | 195 | -86.17 | 601.53 |
| KCl | 500 | 729.33 | 174.35 | 195 | 385.48 | 1073.18 |
| LB | 1000 | 693.30 | 174.35 | 195 | 349.45 | 1037.15 |
| M9 | 1000 | 379.10 | 174.35 | 195 | 35.25 | 722.95 |
| KCl | 1000 | 518.30 | 174.35 | 195 | 174.45 | 862.15 |

**Table S4**

Table S4: Estimated marginal means (emmeans) of bioluminescence responses (B/B_0_, %) across fluoride concentrations and cell preparation conditions for *P. fluorescens* 8866 Tn5 *lux*CDABE.

| Strain: *P. fluorescens* 8866 Tn5 *lux*CDABE | | | | | | |
| --- | --- | --- | --- | --- | --- | --- |
| Condition | **Fluoride (mg L^-1^)** | **emmean** | **SE** | **df** | **lower.CL** | **upper.CL** |
| LB | 0 | 196.82 | 32.18 | 195 | 133.35 | 260.29 |
| M9 | 0 | 165.48 | 32.18 | 195 | 102.01 | 228.95 |
| KCl | 0 | 133.77 | 32.18 | 195 | 70.30 | 197.24 |
| LB | 0.25 | 195.84 | 32.18 | 195 | 132.37 | 259.31 |
| M9 | 0.25 | 164.85 | 32.18 | 195 | 101.38 | 228.32 |
| KCl | 0.25 | 143.22 | 32.18 | 195 | 79.75 | 206.69 |
| LB | 0.5 | 189.01 | 32.18 | 195 | 125.53 | 252.48 |
| M9 | 0.5 | 166.28 | 32.18 | 195 | 102.81 | 229.75 |
| KCl | 0.5 | 140.00 | 32.18 | 195 | 76.53 | 203.47 |
| LB | 1.25 | 189.09 | 32.18 | 195 | 125.62 | 252.56 |
| M9 | 1.25 | 167.66 | 32.18 | 195 | 104.19 | 231.13 |
| KCl | 1.25 | 152.68 | 32.18 | 195 | 89.21 | 216.15 |
| LB | 2.5 | 188.40 | 32.18 | 195 | 124.93 | 251.87 |
| M9 | 2.5 | 166.34 | 32.18 | 195 | 102.87 | 229.81 |
| KCl | 2.5 | 133.05 | 32.18 | 195 | 69.58 | 196.52 |
| LB | 5 | 187.53 | 32.18 | 195 | 124.06 | 251.00 |
| M9 | 5 | 155.91 | 32.18 | 195 | 92.44 | 219.38 |
| KCl | 5 | 131.72 | 32.18 | 195 | 68.25 | 195.19 |
| LB | 12.5 | 189.50 | 32.18 | 195 | 126.03 | 252.97 |
| M9 | 12.5 | 164.49 | 32.18 | 195 | 101.02 | 227.96 |
| KCl | 12.5 | 134.33 | 32.18 | 195 | 70.86 | 197.80 |
| LB | 25 | 192.05 | 32.18 | 195 | 128.58 | 255.52 |
| M9 | 25 | 162.36 | 32.18 | 195 | 98.89 | 225.83 |
| KCl | 25 | 125.80 | 32.18 | 195 | 62.33 | 189.27 |
| LB | 50 | 185.82 | 32.18 | 195 | 122.35 | 249.29 |
| M9 | 50 | 159.08 | 32.18 | 195 | 95.61 | 222.55 |
| KCl | 50 | 136.86 | 32.18 | 195 | 73.39 | 200.33 |
| LB | 125 | 195.75 | 32.18 | 195 | 132.28 | 259.22 |
| M9 | 125 | 169.60 | 32.18 | 195 | 106.13 | 233.07 |
| KCl | 125 | 98.92 | 32.18 | 195 | 35.45 | 162.39 |
| LB | 250 | 196.07 | 32.18 | 195 | 132.60 | 259.54 |
| M9 | 250 | 174.28 | 32.18 | 195 | 110.81 | 237.75 |
| KCl | 250 | 65.17 | 32.18 | 195 | 1.70 | 128.64 |
| LB | 500 | 238.25 | 32.18 | 195 | 174.78 | 301.72 |
| M9 | 500 | 151.64 | 32.18 | 195 | 88.17 | 215.11 |
| KCl | 500 | 43.50 | 32.18 | 195 | -19.97 | 106.97 |
| LB | 1000 | 301.88 | 32.18 | 195 | 238.41 | 365.35 |
| M9 | 1000 | 83.09 | 32.18 | 195 | 19.62 | 146.56 |
| KCl | 1000 | 25.41 | 32.18 | 195 | -38.06 | 88.88 |

**Table S5**

Table S5: Estimated marginal means (emmeans) of bioluminescence responses (B/B_0_, %) across fluoride concentrations and cell preparation conditions for *E. coli* HB101 pUCD607.

| Strain: *E. coli* HB101 pUCD607 | | | | | | |
| --- | --- | --- | --- | --- | --- | --- |
| Condition | **Fluoride (mg L^-1^)** | **emmean** | **SE** | **df** | **lower.CL** | **upper.CL** |
| LB | 0 | 152.55 | 14.74 | 195 | 123.48 | 181.62 |
| M9 | 0 | 99.72 | 14.74 | 195 | 70.65 | 128.80 |
| KCl | 0 | 83.57 | 14.74 | 195 | 54.50 | 112.64 |
| LB | 0.25 | 150.96 | 14.74 | 195 | 121.88 | 180.03 |
| M9 | 0.25 | 101.01 | 14.74 | 195 | 71.94 | 130.09 |
| KCl | 0.25 | 90.32 | 14.74 | 195 | 61.25 | 119.40 |
| LB | 0.5 | 149.55 | 14.74 | 195 | 120.48 | 178.62 |
| M9 | 0.5 | 100.78 | 14.74 | 195 | 71.71 | 129.85 |
| KCl | 0.5 | 89.80 | 14.74 | 195 | 60.73 | 118.87 |
| LB | 1.25 | 150.41 | 14.74 | 195 | 121.34 | 179.49 |
| M9 | 1.25 | 101.15 | 14.74 | 195 | 72.08 | 130.22 |
| KCl | 1.25 | 98.70 | 14.74 | 195 | 69.62 | 127.77 |
| LB | 2.5 | 155.78 | 14.74 | 195 | 126.71 | 184.86 |
| M9 | 2.5 | 103.35 | 14.74 | 195 | 74.28 | 132.43 |
| KCl | 2.5 | 96.30 | 14.74 | 195 | 67.22 | 125.37 |
| LB | 5 | 146.94 | 14.74 | 195 | 117.87 | 176.02 |
| M9 | 5 | 101.80 | 14.74 | 195 | 72.73 | 130.87 |
| KCl | 5 | 91.25 | 14.74 | 195 | 62.17 | 120.32 |
| LB | 12.5 | 144.85 | 14.74 | 195 | 115.77 | 173.92 |
| M9 | 12.5 | 100.61 | 14.74 | 195 | 71.53 | 129.68 |
| KCl | 12.5 | 92.93 | 14.74 | 195 | 63.86 | 122.01 |
| LB | 25 | 148.40 | 14.74 | 195 | 119.33 | 177.47 |
| M9 | 25 | 101.30 | 14.74 | 195 | 72.23 | 130.37 |
| KCl | 25 | 98.53 | 14.74 | 195 | 69.46 | 127.60 |
| LB | 50 | 149.71 | 14.74 | 195 | 120.63 | 178.78 |
| M9 | 50 | 106.05 | 14.74 | 195 | 76.97 | 135.12 |
| KCl | 50 | 99.52 | 14.74 | 195 | 70.44 | 128.59 |
| LB | 125 | 155.62 | 14.74 | 195 | 126.55 | 184.70 |
| M9 | 125 | 105.04 | 14.74 | 195 | 75.96 | 134.11 |
| KCl | 125 | 95.67 | 14.74 | 195 | 66.59 | 124.74 |
| LB | 250 | 161.29 | 14.74 | 195 | 132.22 | 190.36 |
| M9 | 250 | 103.49 | 14.74 | 195 | 74.42 | 132.56 |
| KCl | 250 | 80.30 | 14.74 | 195 | 51.22 | 109.37 |
| LB | 500 | 171.41 | 14.74 | 195 | 142.34 | 200.49 |
| M9 | 500 | 108.95 | 14.74 | 195 | 79.88 | 138.03 |
| KCl | 500 | 64.11 | 14.74 | 195 | 35.03 | 93.18 |
| LB | 1000 | 173.16 | 14.74 | 195 | 144.08 | 202.23 |
| M9 | 1000 | 130.89 | 14.74 | 195 | 101.82 | 159.96 |
| KCl | 1000 | 49.94 | 14.74 | 195 | 20.86 | 79.01 |

**Table S6**

Table S6: Tukey-adjusted pairwise comparisons between cell preparation conditions at each fluoride concentration for *P. putida* F1 Tn5 *lux*CDABE. Significant effects (*p* < 0.05) are shown in bold.

| Strain: *P. putida* F1 Tn5 *lux*CDABE | | | | | | |
| --- | --- | --- | --- | --- | --- | --- |
| Comparison | **Fluoride (mg L^-1^)** | **Estimate** | **SE** | **df** | **t ratio** | **Adjusted**  ***p* value** |
| LB - M9 | 0 | 15.82 | 12.14 | 195 | 1.30 | 0.395 |
| LB - KCl | 0 | 23.00 | 12.14 | 195 | 1.90 | 0.143 |
| M9 - KCl | 0 | 7.19 | 12.14 | 195 | 0.59 | 0.825 |
| LB - M9 | 0.25 | 15.75 | 12.14 | 195 | 1.30 | 0.398 |
| LB - KCl | 0.25 | 18.19 | 12.14 | 195 | 1.50 | 0.294 |
| M9 - KCl | 0.25 | 2.43 | 12.14 | 195 | 0.20 | 0.978 |
| LB - M9 | 0.5 | 19.76 | 12.14 | 195 | 1.63 | 0.236 |
| LB - KCl | 0.5 | 24.24 | 12.14 | 195 | 2.00 | 0.115 |
| M9 - KCl | 0.5 | 4.49 | 12.14 | 195 | 0.37 | 0.927 |
| LB - M9 | 1.25 | 17.46 | 12.14 | 195 | 1.44 | 0.323 |
| LB - KCl | 1.25 | 20.58 | 12.14 | 195 | 1.70 | 0.209 |
| M9 - KCl | 1.25 | 3.11 | 12.14 | 195 | 0.26 | 0.964 |
| LB - M9 | 2.5 | 23.01 | 12.14 | 195 | 1.90 | 0.143 |
| LB - KCl | 2.5 | 26.67 | 12.14 | 195 | 2.20 | 0.074 |
| M9 - KCl | 2.5 | 3.66 | 12.14 | 195 | 0.30 | 0.951 |
| LB - M9 | 5 | 21.93 | 12.14 | 195 | 1.81 | 0.170 |
| LB - KCl | 5 | 27.11 | 12.14 | 195 | 2.23 | 0.068 |
| M9 - KCl | 5 | 5.19 | 12.14 | 195 | 0.43 | 0.904 |
| LB - M9 | 12.5 | 26.41 | 12.14 | 195 | 2.18 | 0.078 |
| LB - KCl | 12.5 | 23.97 | 12.14 | 195 | 1.98 | 0.121 |
| M9 - KCl | 12.5 | -2.44 | 12.14 | 195 | -0.20 | 0.978 |
| LB - M9 | 25 | 27.80 | 12.14 | 195 | 2.29 | 0.059 |
| LB - KCl | 25 | 27.84 | 12.14 | 195 | 2.29 | 0.059 |
| M9 - KCl | 25 | 0.04 | 12.14 | 195 | 0.00 | 1.000 |
| LB - M9 | 50 | 27.23 | 12.14 | 195 | 2.24 | 0.066 |
| LB - KCl | 50 | 23.37 | 12.14 | 195 | 1.93 | 0.134 |
| M9 - KCl | 50 | -3.86 | 12.14 | 195 | -0.32 | 0.946 |
| LB - M9 | 125 | 26.44 | 12.14 | 195 | 2.18 | 0.077 |
| LB - KCl | 125 | 16.00 | 12.14 | 195 | 1.32 | 0.387 |
| M9 - KCl | 125 | -10.45 | 12.14 | 195 | -0.86 | 0.666 |
| LB - M9 | 250 | 31.19 | 12.14 | 195 | 2.57 | **0.029** |
| LB - KCl | 250 | 7.28 | 12.14 | 195 | 0.60 | 0.820 |
| M9 - KCl | 250 | -23.90 | 12.14 | 195 | -1.97 | 0.122 |
| LB - M9 | 500 | 23.60 | 12.14 | 195 | 1.94 | 0.129 |
| LB - KCl | 500 | 11.57 | 12.14 | 195 | 0.95 | 0.607 |
| M9 - KCl | 500 | -12.03 | 12.14 | 195 | -0.99 | 0.583 |
| LB - M9 | 1000 | -15.41 | 12.14 | 195 | -1.27 | 0.414 |
| LB - KCl | 1000 | 66.57 | 12.14 | 195 | 5.49 | **3.79E-07** |
| M9 - KCl | 1000 | 81.99 | 12.14 | 195 | 6.76 | **4.78E-10** |

**Table S7**

Table S7: Tukey-adjusted pairwise comparisons between cell preparation conditions at each fluoride concentration for *P. putida* 12708 Tn5 *lux*CDABE. Significant effects (*p* < 0.05) are shown in bold.

| Strain: *P. putida* 12708 Tn5 *lux*CDABE | | | | | | |
| --- | --- | --- | --- | --- | --- | --- |
| Comparison | **Fluoride (mg L^-1^)** | **Estimate** | **SE** | **df** | **t ratio** | **Adjusted**  ***p* value** |
| LB - M9 | 0 | 487.62 | 246.56 | 195 | 1.98 | 0.120 |
| LB - KCl | 0 | 586.63 | 246.56 | 195 | 2.38 | **0.048** |
| M9 - KCl | 0 | 99.01 | 246.56 | 195 | 0.40 | 0.915 |
| LB - M9 | 0.25 | 493.48 | 246.56 | 195 | 2.00 | 0.115 |
| LB - KCl | 0.25 | 590.55 | 246.56 | 195 | 2.40 | **0.046** |
| M9 - KCl | 0.25 | 97.07 | 246.56 | 195 | 0.39 | 0.918 |
| LB - M9 | 0.5 | 462.06 | 246.56 | 195 | 1.87 | 0.149 |
| LB - KCl | 0.5 | 566.53 | 246.56 | 195 | 2.30 | 0.058 |
| M9 - KCl | 0.5 | 104.48 | 246.56 | 195 | 0.42 | 0.906 |
| LB - M9 | 1.25 | 488.02 | 246.56 | 195 | 1.98 | 0.120 |
| LB - KCl | 1.25 | 571.59 | 246.56 | 195 | 2.32 | 0.056 |
| M9 - KCl | 1.25 | 83.57 | 246.56 | 195 | 0.34 | 0.939 |
| LB - M9 | 2.5 | 506.90 | 246.56 | 195 | 2.06 | 0.102 |
| LB - KCl | 2.5 | 610.55 | 246.56 | 195 | 2.48 | **0.037** |
| M9 - KCl | 2.5 | 103.65 | 246.56 | 195 | 0.42 | 0.907 |
| LB - M9 | 5 | 503.47 | 246.56 | 195 | 2.04 | 0.105 |
| LB - KCl | 5 | 594.53 | 246.56 | 195 | 2.41 | **0.044** |
| M9 - KCl | 5 | 91.06 | 246.56 | 195 | 0.37 | 0.928 |
| LB - M9 | 12.5 | 496.77 | 246.56 | 195 | 2.01 | 0.111 |
| LB - KCl | 12.5 | 599.99 | 246.56 | 195 | 2.43 | **0.042** |
| M9 - KCl | 12.5 | 103.21 | 246.56 | 195 | 0.42 | 0.908 |
| LB - M9 | 25 | 526.52 | 246.56 | 195 | 2.14 | 0.085 |
| LB - KCl | 25 | 618.63 | 246.56 | 195 | 2.51 | **0.034** |
| M9 - KCl | 25 | 92.11 | 246.56 | 195 | 0.37 | 0.926 |
| LB - M9 | 50 | 574.61 | 246.56 | 195 | 2.33 | 0.054 |
| LB - KCl | 50 | 614.37 | 246.56 | 195 | 2.49 | **0.036** |
| M9 - KCl | 50 | 39.76 | 246.56 | 195 | 0.16 | 0.986 |
| LB - M9 | 125 | 578.19 | 246.56 | 195 | 2.34 | 0.052 |
| LB - KCl | 125 | 584.51 | 246.56 | 195 | 2.37 | **0.049** |
| M9 - KCl | 125 | 6.32 | 246.56 | 195 | 0.03 | 1.000 |
| LB - M9 | 250 | 590.98 | 246.56 | 195 | 2.40 | **0.046** |
| LB - KCl | 250 | 457.68 | 246.56 | 195 | 1.86 | 0.154 |
| M9 - KCl | 250 | -133.30 | 246.56 | 195 | -0.54 | 0.851 |
| LB - M9 | 500 | 594.34 | 246.56 | 195 | 2.41 | **0.044** |
| LB - KCl | 500 | 122.69 | 246.56 | 195 | 0.50 | 0.873 |
| M9 - KCl | 500 | -471.65 | 246.56 | 195 | -1.91 | 0.138 |
| LB - M9 | 1000 | 314.20 | 246.56 | 195 | 1.27 | 0.411 |
| LB - KCl | 1000 | 175.00 | 246.56 | 195 | 0.71 | 0.758 |
| M9 - KCl | 1000 | -139.20 | 246.56 | 195 | -0.56 | 0.839 |

**Table S8**

Table S8: Tukey-adjusted pairwise comparisons between cell preparation conditions at each fluoride concentration for *P. fluorescens* 8866 Tn5 *lux*CDABE. Significant effects (*p* < 0.05) are shown in bold.

| Strain: *P. fluorescens* 8866 Tn5 *lux*CDABE | | | | | | |
| --- | --- | --- | --- | --- | --- | --- |
| Comparison | **Fluoride (mg L^-1^)** | **Estimate** | **SE** | **df** | **t ratio** | **Adjusted**  ***p* value** |
| LB - M9 | 0 | 31.33 | 45.51 | 195 | 0.69 | 0.771 |
| LB - KCl | 0 | 63.05 | 45.51 | 195 | 1.39 | 0.351 |
| M9 - KCl | 0 | 31.72 | 45.51 | 195 | 0.70 | 0.766 |
| LB - M9 | 0.25 | 30.99 | 45.51 | 195 | 0.68 | 0.775 |
| LB - KCl | 0.25 | 52.61 | 45.51 | 195 | 1.16 | 0.481 |
| M9 - KCl | 0.25 | 21.62 | 45.51 | 195 | 0.48 | 0.883 |
| LB - M9 | 0.5 | 22.72 | 45.51 | 195 | 0.50 | 0.872 |
| LB - KCl | 0.5 | 49.01 | 45.51 | 195 | 1.08 | 0.530 |
| M9 - KCl | 0.5 | 26.28 | 45.51 | 195 | 0.58 | 0.832 |
| LB - M9 | 1.25 | 21.43 | 45.51 | 195 | 0.47 | 0.885 |
| LB - KCl | 1.25 | 36.42 | 45.51 | 195 | 0.80 | 0.703 |
| M9 - KCl | 1.25 | 14.99 | 45.51 | 195 | 0.33 | 0.942 |
| LB - M9 | 2.5 | 22.06 | 45.51 | 195 | 0.48 | 0.879 |
| LB - KCl | 2.5 | 55.35 | 45.51 | 195 | 1.22 | 0.445 |
| M9 - KCl | 2.5 | 33.29 | 45.51 | 195 | 0.73 | 0.745 |
| LB - M9 | 5 | 31.63 | 45.51 | 195 | 0.69 | 0.767 |
| LB - KCl | 5 | 55.81 | 45.51 | 195 | 1.23 | 0.439 |
| M9 - KCl | 5 | 24.19 | 45.51 | 195 | 0.53 | 0.856 |
| LB - M9 | 12.5 | 25.01 | 45.51 | 195 | 0.55 | 0.847 |
| LB - KCl | 12.5 | 55.17 | 45.51 | 195 | 1.21 | 0.447 |
| M9 - KCl | 12.5 | 30.16 | 45.51 | 195 | 0.66 | 0.785 |
| LB - M9 | 25 | 29.69 | 45.51 | 195 | 0.65 | 0.791 |
| LB - KCl | 25 | 66.25 | 45.51 | 195 | 1.46 | 0.315 |
| M9 - KCl | 25 | 36.56 | 45.51 | 195 | 0.80 | 0.701 |
| LB - M9 | 50 | 26.74 | 45.51 | 195 | 0.59 | 0.827 |
| LB - KCl | 50 | 48.96 | 45.51 | 195 | 1.08 | 0.530 |
| M9 - KCl | 50 | 22.22 | 45.51 | 195 | 0.49 | 0.877 |
| LB - M9 | 125 | 26.16 | 45.51 | 195 | 0.57 | 0.834 |
| LB - KCl | 125 | 96.83 | 45.51 | 195 | 2.13 | 0.087 |
| M9 - KCl | 125 | 70.68 | 45.51 | 195 | 1.55 | 0.269 |
| LB - M9 | 250 | 21.79 | 45.51 | 195 | 0.48 | 0.881 |
| LB - KCl | 250 | 130.90 | 45.51 | 195 | 2.88 | **0.012** |
| M9 - KCl | 250 | 109.11 | 45.51 | 195 | 2.40 | **0.046** |
| LB - M9 | 500 | 86.62 | 45.51 | 195 | 1.90 | 0.140 |
| LB - KCl | 500 | 194.75 | 45.51 | 195 | 4.28 | **8.69E-05** |
| M9 - KCl | 500 | 108.13 | 45.51 | 195 | 2.38 | **0.048** |
| LB - M9 | 1000 | 218.79 | 45.51 | 195 | 4.81 | **9.09E-06** |
| LB - KCl | 1000 | 276.47 | 45.51 | 195 | 6.07 | **1.92E-08** |
| M9 - KCl | 1000 | 57.68 | 45.51 | 195 | 1.27 | 0.415 |

**Table S9**

Table S9: Tukey-adjusted pairwise comparisons between cell preparation conditions at each fluoride concentration for *E. coli* HB101 pUCD607. Significant effects (*p* < 0.05) are shown in bold.

| Strain: *E. coli* HB101 pUCD607 | | | | | | |
| --- | --- | --- | --- | --- | --- | --- |
| Comparison | **Fluoride (mg L^-1^)** | **Estimate** | **SE** | **df** | **t ratio** | **Adjusted**  ***p* value** |
| LB - M9 | 0 | 52.83 | 20.85 | 195 | 2.53 | **0.032** |
| LB - KCl | 0 | 68.98 | 20.85 | 195 | 3.31 | **0.003** |
| M9 - KCl | 0 | 16.16 | 20.85 | 195 | 0.77 | 0.719 |
| LB - M9 | 0.25 | 49.94 | 20.85 | 195 | 2.40 | **0.046** |
| LB - KCl | 0.25 | 60.63 | 20.85 | 195 | 2.91 | **0.011** |
| M9 - KCl | 0.25 | 10.69 | 20.85 | 195 | 0.51 | 0.865 |
| LB - M9 | 0.5 | 48.77 | 20.85 | 195 | 2.34 | 0.053 |
| LB - KCl | 0.5 | 59.75 | 20.85 | 195 | 2.87 | **0.013** |
| M9 - KCl | 0.5 | 10.98 | 20.85 | 195 | 0.53 | 0.858 |
| LB - M9 | 1.25 | 49.26 | 20.85 | 195 | 2.36 | **0.050** |
| LB - KCl | 1.25 | 51.72 | 20.85 | 195 | 2.48 | **0.037** |
| M9 - KCl | 1.25 | 2.45 | 20.85 | 195 | 0.12 | 0.992 |
| LB - M9 | 2.5 | 52.43 | 20.85 | 195 | 2.51 | **0.034** |
| LB - KCl | 2.5 | 59.49 | 20.85 | 195 | 2.85 | **0.013** |
| M9 - KCl | 2.5 | 7.06 | 20.85 | 195 | 0.34 | 0.939 |
| LB - M9 | 5 | 45.14 | 20.85 | 195 | 2.17 | 0.080 |
| LB - KCl | 5 | 55.70 | 20.85 | 195 | 2.67 | **0.022** |
| M9 - KCl | 5 | 10.55 | 20.85 | 195 | 0.51 | 0.868 |
| LB - M9 | 12.5 | 44.24 | 20.85 | 195 | 2.12 | 0.088 |
| LB - KCl | 12.5 | 51.91 | 20.85 | 195 | 2.49 | **0.036** |
| M9 - KCl | 12.5 | 7.67 | 20.85 | 195 | 0.37 | 0.928 |
| LB - M9 | 25 | 47.10 | 20.85 | 195 | 2.26 | 0.064 |
| LB - KCl | 25 | 49.87 | 20.85 | 195 | 2.39 | **0.046** |
| M9 - KCl | 25 | 2.77 | 20.85 | 195 | 0.13 | 0.990 |
| LB - M9 | 50 | 43.66 | 20.85 | 195 | 2.09 | 0.094 |
| LB - KCl | 50 | 50.19 | 20.85 | 195 | 2.41 | **0.045** |
| M9 - KCl | 50 | 6.53 | 20.85 | 195 | 0.31 | 0.947 |
| LB - M9 | 125 | 50.59 | 20.85 | 195 | 2.43 | **0.043** |
| LB - KCl | 125 | 59.95 | 20.85 | 195 | 2.88 | **0.012** |
| M9 - KCl | 125 | 9.37 | 20.85 | 195 | 0.45 | 0.895 |
| LB - M9 | 250 | 57.80 | 20.85 | 195 | 2.77 | **0.017** |
| LB - KCl | 250 | 80.99 | 20.85 | 195 | 3.88 | **4.10E-04** |
| M9 - KCl | 250 | 23.19 | 20.85 | 195 | 1.11 | 0.508 |
| LB - M9 | 500 | 62.46 | 20.85 | 195 | 3.00 | **0.009** |
| LB - KCl | 500 | 107.31 | 20.85 | 195 | 5.15 | **1.92E-06** |
| M9 - KCl | 500 | 44.85 | 20.85 | 195 | 2.15 | 0.082 |
| LB - M9 | 1000 | 42.27 | 20.85 | 195 | 2.03 | 0.108 |
| LB - KCl | 1000 | 123.22 | 20.85 | 195 | 5.91 | **4.49E-08** |
| M9 - KCl | 1000 | 80.96 | 20.85 | 195 | 3.88 | **4.13E-04** |

**Table S10**

Table S10: One-way ANOVA of biological replicate growth rate values in *lux*-marked *Pseudomonas putida* strains following PFAS exposure. Significant effects (*p* < 0.05) are shown in bold.

| Strain | PFAS compound | Source | df | Sum Sq | Mean Sq | F | *p* value |
| --- | --- | --- | --- | --- | --- | --- | --- |
| *P. putida* 12708 Tn5 *lux*CDABE | PFHxS | Concentration | 3 | 0.303 | 0.101 | 7.49 | **0.010** |
|  |  | Residuals | 8 | 0.108 | 0.014 | - | - |
|  | PFNA | Concentration | 3 | 0.084 | 0.028 | 1.94 | 0.224 |
|  |  | Residuals | 6 | 0.087 | 0.014 | - | - |
|  | PFOA | Concentration | 3 | 0.119 | 0.040 | 12.33 | **0.004** |
|  |  | Residuals | 7 | 0.023 | 0.003 | - | - |
|  | PFOS | Concentration | 3 | 0.131 | 0.044 | 5.22 | **0.033** |
|  |  | Residuals | 7 | 0.059 | 0.008 | - | - |
| *P. putida* F1 Tn5 *lux*CDABE | PFHxS | Concentration | 3 | 0.030 | 0.010 | 30.88 | **0.003** |
|  |  | Residuals | 4 | 0.001 | 0.000 | - | - |
|  | PFNA | Concentration | 3 | 0.112 | 0.037 | 15.62 | **0.001** |
|  |  | Residuals | 8 | 0.019 | 0.002 | - | - |
|  | PFOA | Concentration | 3 | 0.010 | 0.003 | 1.07 | 0.457 |
|  |  | Residuals | 4 | 0.013 | 0.003 | - | - |

**Table S11**

Table S11: Dunnett-adjusted post hoc comparisons of individual biological replicate growth rates relative to the untreated control in *lux*-marked *Pseudomonas putida* strains following PFAS exposure.

| Strain | PFAS compound | Comparison | Estimate | SE | df | t value | Adjusted *p* value |
| --- | --- | --- | --- | --- | --- | --- | --- |
| *P. putida* 12708 Tn5 *lux*CDABE | PFHxS | 0.1 vs control | 0.066 | 0.095 | 8 | 0.70 | 0.808 |
|  |  | 1 vs control | 0.000 | 0.095 | 8 | 0.00 | 1.000 |
|  |  | 10 vs control | 0.384 | 0.095 | 8 | 4.05 | **0.010** |
|  | PFNA | 0.1 vs control | -0.064 | 0.110 | 6 | -0.58 | 0.864 |
|  |  | 1 vs control | -0.195 | 0.098 | 6 | -1.98 | 0.218 |
|  |  | 10 vs control | -0.216 | 0.110 | 6 | -1.97 | 0.221 |
|  | PFOA | 0.1 vs control | 0.174 | 0.046 | 7 | 3.76 | **0.018** |
|  |  | 1 vs control | 0.134 | 0.046 | 7 | 2.88 | 0.059 |
|  |  | 10 vs control | 0.308 | 0.052 | 7 | 5.95 | **0.002** |
|  | PFOS | 0.1 vs control | 0.105 | 0.084 | 7 | 1.25 | 0.502 |
|  |  | 1 vs control | -0.165 | 0.084 | 7 | -1.97 | 0.207 |
|  |  | 10 vs control | -0.121 | 0.084 | 7 | -1.44 | 0.405 |
| *P. putida* F1 Tn5 *lux*CDABE | PFHxS | 0.1 vs control | -0.113 | 0.018 | 4 | -6.25 | **0.008** |
|  |  | 1 vs control | -0.153 | 0.018 | 4 | -8.49 | **0.003** |
|  |  | 10 vs control | -0.146 | 0.018 | 4 | -8.09 | **0.003** |
|  | PFNA | 0.1 vs control | -0.244 | 0.040 | 8 | -6.11 | **0.001** |
|  |  | 1 vs control | -0.217 | 0.040 | 8 | -5.45 | **0.002** |
|  |  | 10 vs control | -0.198 | 0.040 | 8 | -4.97 | **0.003** |
|  | PFOA | 0.1 vs control | 0.090 | 0.057 | 4 | 1.58 | 0.392 |
|  |  | 1 vs control | 0.070 | 0.057 | 4 | 1.22 | 0.552 |
|  |  | 10 vs control | 0.021 | 0.057 | 4 | 0.37 | 0.945 |

**Table S12**

Table S12: Diagnostic assessment of one-way ANOVA assumptions for growth rate values from biological replicates in *lux*-marked *Pseudomonas putida* strains following PFAS exposure.

| Strain | PFAS compound | Residuals (n) | Shapiro-Wilk *p* |
| --- | --- | --- | --- |
| *P. putida* 12708 Tn5 *lux*CDABE | PFHxS | 12 | 0.466 |
|  | PFNA | 10 | 0.382 |
|  | PFOA | 11 | 0.306 |
|  | PFOS | 11 | 0.022 |
| *P. putida* F1 Tn5 *lux*CDABE | PFHxS | 8 | 0.133 |
|  | PFNA | 12 | 0.187 |
|  | PFOA | 8 | 0.787 |

**Table S13**

Table S13: One-way ANOVA of replicate-level peak bioluminescence inhibition in *P. putida* F1 Tn5 *luxCDABE* following PFAS exposure at 6 h.

| PFAS compound | Source | df | Sum Sq | Mean Sq | F | *p* value |
| --- | --- | --- | --- | --- | --- | --- |
| PFOA | Concentration | 3 | 1779.8 | 593.3 | 8.92 | **0.030** |
| PFOA | Residuals | 4 | 266.0 | 66.5 | - | - |
| PFHxS | Concentration | 3 | 9892.6 | 3297.5 | 4.68 | 0.085 |
| PFHxS | Residuals | 4 | 2820.1 | 705.0 | - | - |
| PFNA | Concentration | 3 | 13373.9 | 4458.0 | 5.94 | **0.020** |
| PFNA | Residuals | 8 | 6000.8 | 750.1 | - | - |

**Table S14**

Table S14: Dunnett-adjusted post hoc comparisons of replicate-level peak bioluminescence inhibition (t = 6 h) for each PFAS compound and concentration relative to the untreated control (0 mg L^-1^) in *P. putida* F1 Tn5 *luxCDABE*. Significant differences (*p* < 0.05) are shown in bold.

| PFAS compound | Comparison | Estimate | SE | df | t value | Adjusted *p* value |
| --- | --- | --- | --- | --- | --- | --- |
| PFOA | 0.1 vs control | 4.81 | 8.15 | 4 | 0.59 | 0.865 |
| PFOA | 1 vs control | 22.44 | 8.15 | 4 | 2.75 | 0.119 |
| PFOA | 10 vs control | 37.62 | 8.15 | 4 | 4.61 | **0.024** |
| PFHxS | 0.1 vs control | -39.55 | 26.55 | 4 | -1.49 | 0.427 |
| PFHxS | 1 vs control | 58.53 | 26.55 | 4 | 2.20 | 0.207 |
| PFHxS | 10 vs control | -3.74 | 26.55 | 4 | -0.14 | 0.992 |
| PFNA | 0.1 vs control | 87.14 | 22.36 | 8 | 3.90 | **0.012** |
| PFNA | 1 vs control | 72.11 | 22.36 | 8 | 3.22 | **0.032** |
| PFNA | 10 vs control | 40.51 | 22.36 | 8 | 1.81 | 0.247 |

**Table S15**

Table S15: Diagnostic assessment of one-way ANOVA assumptions for replicate-level peak bioluminescence inhibition in *P. putida* F1 Tn5 *luxCDABE* at 6 h. Values reported include the number of residual observations and Shapiro–Wilk test *p* values used to assess residual normality. Values of *p* > 0.05 indicate no evidence of significant deviation from normality.

| PFAS compound | Residuals (n) | Shapiro-Wilk *p* |
| --- | --- | --- |
| PFOA | 8 | 0.600 |
| PFHxS | 8 | 0.853 |
| PFNA | 12 | 0.332 |

**Table S16**

Table S16: One-way ANOVA of replicate-level peak bioluminescence inhibition in *P. putida* 12708 Tn5 *luxCDABE* following PFAS exposure across the peak bioluminescence window between 7.5-12 h.

| PFAS compound | Source | df | Sum Sq | Mean Sq | F | *p* value |
| --- | --- | --- | --- | --- | --- | --- |
| PFOA | Concentration | 4 | 40076.9 | 10019.2 | 6.91 | **0.006** |
| PFOA | Residuals | 10 | 14502.2 | 1450.2 | - | - |
| PFHxS | Concentration | 4 | 15009.8 | 3752.4 | 2.68 | 0.094 |
| PFHxS | Residuals | 10 | 13985.5 | 1398.5 | - | - |
| PFNA | Concentration | 4 | 128297.4 | 32074.4 | 1.06 | 0.424 |
| PFNA | Residuals | 10 | 301631.5 | 30163.2 | - | **-** |
| PFOS | Concentration | 4 | 20871.8 | 5217.9 | 0.87 | 0.513 |
| PFOS | Residuals | 10 | 59766.0 | 5976.6 | - | - |

**Table S17**

Table S17: Dunnett-adjusted post hoc comparisons of replicate-level peak bioluminescence inhibition (t = 7.5-12 h) for each PFAS compound and concentration relative to the untreated control (0 mg L^-1^) in *P. putida* 12708 Tn5 *luxCDABE*. Significant differences (*p* < 0.05) are shown in bold.

| PFAS compound | Comparison | Estimate | SE | df | t value | Adjusted *p* value |
| --- | --- | --- | --- | --- | --- | --- |
| PFOA | 0.1 vs control | -80.80 | 31.09 | 10 | -2.60 | 0.084 |
| PFOA | 1 vs control | -27.33 | 31.09 | 10 | -0.88 | 0.766 |
| PFOA | 10 vs control | 11.44 | 31.09 | 10 | 0.37 | 0.966 |
| PFOA | 100 vs control | -125.97 | 31.09 | 10 | -4.05 | **0.008** |
| PFHxS | 0.1 vs control | -15.65 | 30.53 | 10 | -0.51 | 0.928 |
| PFHxS | 1 vs control | -25.26 | 30.53 | 10 | -0.83 | 0.793 |
| PFHxS | 10 vs control | -27.25 | 30.53 | 10 | -0.89 | 0.759 |
| PFHxS | 100 vs control | -92.37 | 30.53 | 10 | -3.02 | **0.042** |
| PFNA | 0.1 vs control | -57.23 | 141.81 | 10 | -0.40 | 0.958 |
| PFNA | 1 vs control | 5.88 | 141.81 | 10 | 0.04 | 1.000 |
| PFNA | 10 vs control | -56.44 | 141.81 | 10 | -0.40 | 0.960 |
| PFNA | 100 vs control | -248.24 | 141.81 | 10 | -1.75 | 0.303 |
| PFOS | 0.1 vs control | 11.52 | 63.12 | 10 | 0.18 | 0.994 |
| PFOS | 1 vs control | 15.03 | 63.12 | 10 | 0.24 | 0.988 |
| PFOS | 10 vs control | 88.03 | 63.12 | 10 | 1.39 | 0.474 |
| PFOS | 100 vs control | -22.98 | 63.12 | 10 | -0.36 | 0.967 |

**Table S18**

Table S18: Diagnostic assessment of one-way ANOVA assumptions for replicate-level peak bioluminescence inhibition in *P. putida* 12708 Tn5 *luxCDABE* between 7.5-12 h. Values reported include the number of residual observations and Shapiro–Wilk test *p* values used to assess residual normality. Values of *p* > 0.05 indicate no evidence of significant deviation from normality.

| PFAS compound | Residuals (n) | Shapiro-Wilk *p* |
| --- | --- | --- |
| PFOA | 15 | 0.136 |
| PFHxS | 15 | 0.112 |
| PFNA | 15 | 0.098 |
| PFOS | 15 | 0.056 |

**Table S19**

Table S19: One-way ANOVA comparing replicate-level peak bioluminescence inhibition between PFAS compounds within each concentration in *P. putida* F1 Tn5 *luxCDABE* at 6 h. Significant effects (*p* < 0.05) are shown in bold.

| PFAS  concentration | Source | df | Sum Sq | Mean Sq | F | *p* value |
| --- | --- | --- | --- | --- | --- | --- |
| 0.1 | Compound | 2 | 20691.3 | 10345.6 | 129.58 | **0.0002** |
| 0.1 | Residuals | 4 | 319.4 | 79.8 |  |  |
| 1 | Compound | 2 | 3017.2 | 1508.6 | 1.31 | 0.364 |
| 1 | Residuals | 4 | 4593.3 | 1148.3 |  |  |
| 10 | Compound | 2 | 2662.3 | 1331.2 | 1.28 | 0.373 |
| 10 | Residuals | 4 | 4174.2 | 1043.6 |  |  |

**Table S20**

Table S20: Tukey-adjusted pairwise comparisons of replicate-level peak bioluminescence inhibition between PFAS compounds within each concentration in *P. putida* F1 Tn5 *luxCDABE* at 6 h. Significant differences (*p* < 0.05) are shown in bold.

| PFAS  concentration | Comparison | Estimate | SE | df | t value | Adjusted  *p* value |
| --- | --- | --- | --- | --- | --- | --- |
| 0.1 | PFOA - PFHxS | 44.36 | 8.94 | 4 | 4.96 | 0.017 |
| 0.1 | PFOA - PFNA | -82.33 | 8.16 | 4 | -10.09 | **0.001** |
| 0.1 | PFHxS - PFNA | -126.69 | 8.16 | 4 | -15.53 | **0.0002** |
| 1 | PFOA - PFHxS | -36.10 | 33.89 | 4 | -1.07 | 0.581 |
| 1 | PFOA - PFNA | -49.67 | 30.93 | 4 | -1.61 | 0.343 |
| 1 | PFHxS - PFNA | -13.58 | 30.93 | 4 | -0.44 | 0.902 |
| 10 | PFOA - PFHxS | 41.36 | 32.30 | 4 | 1.28 | 0.475 |
| 10 | PFOA - PFNA | -2.89 | 29.49 | 4 | -0.10 | 0.995 |
| 10 | PFHxS - PFNA | -44.24 | 29.49 | 4 | -1.50 | 0.382 |

**Table S21**

Table S21: Diagnostic assessment of one-way ANOVA assumptions for replicate-level peak bioluminescence inhibition between PFAS compounds within each concentration in *P. putida* F1 Tn5 *luxCDABE* at 6 h.

| Concentration (mg L^-1^) | Residuals (n) | Shapiro-Wilk *p* |
| --- | --- | --- |
| 0.1 | 7 | 0.864 |
| 1 | 7 | 0.415 |
| 10 | 7 | 0.499 |

**Table S22**

Table S22: One-way ANOVA comparing replicate-level peak bioluminescence inhibition between PFAS compounds within each concentration in *P. putida* 12708 Tn5 *luxCDABE* across the peak luminescence window (t = 7.5-12 h). Significant effects (*p* < 0.05) are shown in bold.

| PFAS  concentration | Source | df | Sum Sq | Mean Sq | F | *p* value |
| --- | --- | --- | --- | --- | --- | --- |
| 0.1 | Compound | 3 | 15388.8 | 5129.6 | 1.27 | 0.347 |
| 0.1 | Residuals | 8 | 32186.9 | 4023.4 |  |  |
| 1 | Compound | 3 | 4183.6 | 1394.5 | 0.19 | 0.903 |
| 1 | Residuals | 8 | 59805.4 | 7475.7 |  |  |
| 10 | Compound | 3 | 35238.1 | 11746.0 | 1.18 | 0.378 |
| 10 | Residuals | 8 | 79964.6 | 9995.6 |  |  |
| 100 | Compound | 3 | 79905.0 | 26635.0 | 0.98 | 0.450 |
| 100 | Residuals | 8 | 217928.3 | 27241.0 |  |  |

**Table S23**

Table S23: Tukey-adjusted pairwise comparisons of replicate-level peak bioluminescence inhibition between PFAS compounds within each concentration in *P. putida* 12708 Tn5 *luxCDABE* between 7.5-12 h. Significant differences (*p* < 0.05) are shown in bold.

| PFAS  concentration | Comparison | Estimate | SE | df | t value | Adjusted  *p* value |
| --- | --- | --- | --- | --- | --- | --- |
| 0.1 | PFOA - PFHxS | -65.15 | 51.79 | 8 | -1.26 | 0.611 |
| 0.1 | PFOA - PFNA | -23.57 | 51.79 | 8 | -0.46 | 0.967 |
| 0.1 | PFOA - PFOS | -92.32 | 51.79 | 8 | -1.78 | 0.347 |
| 0.1 | PFHxS - PFNA | 41.59 | 51.79 | 8 | 0.80 | 0.851 |
| 0.1 | PFHxS - PFOS | -27.17 | 51.79 | 8 | -0.52 | 0.951 |
| 0.1 | PFNA - PFOS | -68.76 | 51.79 | 8 | -1.33 | 0.572 |
| 1 | PFOA - PFHxS | -2.06 | 70.60 | 8 | -0.03 | 1.000 |
| 1 | PFOA - PFNA | -33.21 | 70.60 | 8 | -0.47 | 0.964 |
| 1 | PFOA - PFOS | -42.36 | 70.60 | 8 | -0.60 | 0.929 |
| 1 | PFHxS - PFNA | -31.14 | 70.60 | 8 | -0.44 | 0.970 |
| 1 | PFHxS - PFOS | -40.29 | 70.60 | 8 | -0.57 | 0.938 |
| 1 | PFNA - PFOS | -9.15 | 70.60 | 8 | -0.13 | 0.999 |
| 10 | PFOA - PFHxS | 38.69 | 81.63 | 8 | 0.47 | 0.963 |
| 10 | PFOA - PFNA | 67.88 | 81.63 | 8 | 0.83 | 0.838 |
| 10 | PFOA - PFOS | -76.59 | 81.63 | 8 | -0.94 | 0.786 |
| 10 | PFHxS - PFNA | 29.19 | 81.63 | 8 | 0.36 | 0.983 |
| 10 | PFHxS - PFOS | -115.28 | 81.63 | 8 | -1.41 | 0.526 |
| 10 | PFNA - PFOS | -144.47 | 81.63 | 8 | -1.77 | 0.352 |
| 100 | PFOA - PFHxS | -33.61 | 134.76 | 8 | -0.25 | 0.994 |
| 100 | PFOA - PFNA | 122.27 | 134.76 | 8 | 0.91 | 0.802 |
| 100 | PFOA - PFOS | -102.99 | 134.76 | 8 | -0.76 | 0.868 |
| 100 | PFHxS - PFNA | 155.87 | 134.76 | 8 | 1.16 | 0.668 |
| 100 | PFHxS - PFOS | -69.39 | 134.76 | 8 | -0.51 | 0.953 |
| 100 | PFNA - PFOS | -225.26 | 134.76 | 8 | -1.67 | 0.396 |

**Table S24**

Table S24: Diagnostic assessment of one-way ANOVA assumptions for replicate-level peak bioluminescence inhibition between PFAS compounds within each concentration in *P. putida* 12708 Tn5 *luxCDABE* between 7.5-12 h.

| Concentration (mg L^-1^) | Residuals (n) | Shapiro-Wilk *p* |
| --- | --- | --- |
| 0.1 | 12 | 0.577 |
| 1 | 12 | 0.291 |
| 10 | 12 | 0.003 |
| 100 | 12 | 0.038 |
